# Dynamic Confinement Approach for High Metal Loading Single‐Atom Catalysts Based on Covalent Organic Frameworks

**DOI:** 10.1002/anie.202522238

**Published:** 2026-01-01

**Authors:** Kyung Seob Song, Murad Najafov, José Manuel González Acosta, Andrea Ruiz Ferrando, Stephan Pollitt, Patrick W. Fritz, Timur Ashirov, Krzysztof Piech, Felipe Gándara, Maarten Nachtegaal, Núria López, Ali Coskun

**Affiliations:** ^1^ Department of Chemistry University of Fribourg Chemin du Musee 9 Fribourg 1700 Switzerland; ^2^ National Centre of Competence in Research (NCCR) Catalysis University of Fribourg Fribourg 1700 Switzerland; ^3^ Institut Català d'Investigació Química (ICIQ‐CERCA) Av. Països Catalans, 16 – Tarragona 43007 Spain; ^4^ Department of Physical and Inorganic Chemistry Universitat Rovira i Virgili Marcel·lí Domingo s/n Tarragona 43007 Spain; ^5^ Laboratory for Synchrotron Radiation and Femtochemistry (LSF) Paul Scherrer Institute Forschungsstrasse 111 Villigen 5232 Switzerland; ^6^ Department of New Architectures in Materials Chemistry Materials Science Institute of Madrid—CSIC Sor Juana Inés de la Cruz 3 Madrid 28049 Spain

**Keywords:** Flow chemistry, Heterogeneous catalysis, Platinum group metals, Porous materials

## Abstract

Single‐atom catalysts (SACs) offer stable, well‐defined active sites by anchoring individual metal atoms on stable organic or inorganic supports, though achieving high metal loadings without clustering or leaching remains a major challenge. Here, we report a synthetic strategy for developing ultra‐high metal loading SACs based on palladium polyphthalocyanine covalent organic frameworks (COFs) synthesized via a mixed metal ionothermal approach, which involves the cyclization of tetracyanobenzene and tetracyanopyrazine as precursors in molten salt mixtures of PdCl_2_/ZnCl_2_ or PdCl_2_/ZnCl_2_/NaCl. This approach effectively combines the formation of crystalline polymeric hosts with metal impregnation in a single step, yielding COFs with atomically distributed Pd ions and metal contents of up to 22.2 wt%. Theoretical simulations reveal that the crystalline framework dynamically confines Pd atoms between different binding sites within the pores, preventing dimerization and ensuring long‐term catalyst stability. The synthesized catalysts were evaluated under continuous flow conditions, exhibiting stable performance with yields as high as 90% and maintaining stability over a 24 h time‐on‐stream under low‐conversion conditions. These results establish a new benchmark for SACs and underscore the importance of dynamic confinement approach in achieving high metal loadings on crystalline organic supports.

## Graphical abstract

We introduced a new synthetic strategy for the preparation of high metal loading crystalline single‐atom catalysts based on covalent organic frameworks with atomically distributed Pd ions and metal contents of up to 22.2 wt%. Theoretical simulations reveal that the crystalline framework dynamically confines Pd atoms between different binding sites within the pores, ensuring long‐term catalyst stability under practical conditions.

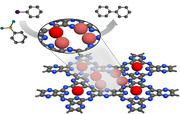

## Introduction

Heterogeneous single‐atom catalysts (SACs) comprising isolated individual atoms dispersed on a support material represent a significant advance in catalysis over conventional catalysts, which rely on the presence of metal clusters and/or nanoparticles.^[^ ^1^, ^2^, ^3^ ^]^ Owing to their maximized atomic efficiency and unique electronic environments, SACs have already been employed in a wide range of reactions, including oxidation processes,^[^ ^4^ ^]^ hydrogenations,^[^ ^5^ ^]^ photocatalysis,^[^ ^6^ ^]^ electrocatalysis,^[^ ^7^ ^]^ and cross‐coupling reactions.^[^ ^8^ ^]^ The synthesis of heterogeneous SACs, including platinum group metals such as rhodium, platinum, and palladium as well as transition metals like nickel, iron, copper, and cobalt, can be realized through various techniques including wet‐impregnation,^[^ ^9^ ^]^ dry impregnation,^[^ ^10^, ^11^ ^]^ ion‐exchange,^[^ ^12^ ^]^ atomic layer deposition (ALD)^[^ ^13^ ^]^ and pyrolysis of metal–organic frameworks (MOFs).^[^ ^14^ ^]^ Precise control over the synthetic conditions is, however, critical to achieving atomically dispersed species, as the aggregation of atoms into nanoparticles can occur readily, which naturally limits the metal loadings in SACs. Whereas metals and oxide materials have been primarily used as supports, more recently, organic supports, i.e., graphitic carbon nitride, g‐C_3_N_4_, and N‐doped carbons, have emerged as promising alternatives and have been employed as supports for the development of SACs for cross‐coupling reactions. SACs featuring organic supports such as g‐C_3_N_4_ and N‐doped carbon synthesized through the wet‐impregnation approach, however, are frequently limited to low metal contents. This limitation is mainly due to the poor interaction between the metal precursors and the support, which often causes the metal atoms to cluster during synthesis or post‐treatment. Likewise, although the incipient wetness impregnation method allows for more controlled dispersion of the metal, it is still limited to low metal loadings because of the limited pore volume and weak binding between the metal species and the organic framework.^[^ ^15^ ^]^ As an alternative, the pyrolysis of MOFs has emerged as an interesting approach to form N‐doped carbons with high Co loadings exceeding 4 wt% ^[^ ^16^ ^]^ or high Fe loadings of about 7 wt% ^[^ ^17^ ^]^ and used as highly efficient electrocatalysts for the oxygen reduction reaction.^[^ ^18^ ^]^ More recently, the wet‐impregnation method has been complemented to afford materials with high densities of surface single atoms. In this direction, Hai et al. have introduced an elegant approach to achieving scalable, ultra‐high metal density SACs with Pd metal loadings for g‐C_3_N_4_ above 20 wt% by combining wet‐impregnation with a two‐step annealing process, the catalytic performance of these particular SACs, however have not been investigated.^[^ ^19^ ^]^ The fact that these high metal loadings can be achieved with g‐C_3_N_4_ and/or N‐doped carbons showcases the potential of organic supports in the further development of SACs. The lack of precise control over the nature of metal coordination sites, in other words, binding site heterogeneity in these supports, presents critical challenges to understanding the catalytic activity of SACs.

The development of new synthetic strategies and, in particular, crystalline organic supports will provide a deeper understanding of the fundamental principles governing SACs. In this context, covalent organic frameworks (COFs) are promising candidates owing to their ordered structures, high porosity, and tunability.^[^ ^20^ ^]^ Notably, the precise control of their structure enables the creation of well‐defined catalytic sites for metal anchoring, their uniform distribution, as well as accessibility owing to their high surface areas. So far, the reported SACs based on COFs also exhibited low metal contents, relying on either pre‐functionalization of the linker with the respective metal complex^[^ ^21^ ^]^ or wet‐impregnation.^[^ ^11^ ^]^ More recently, interlayer coordination of PdCl_2_ onto imine nitrogen of a COF enabled Pd single atom loadings of 18.1 wt% and was used in gas‐phase catalytic applications.^[^ ^22^ ^]^

**Figure 1:**
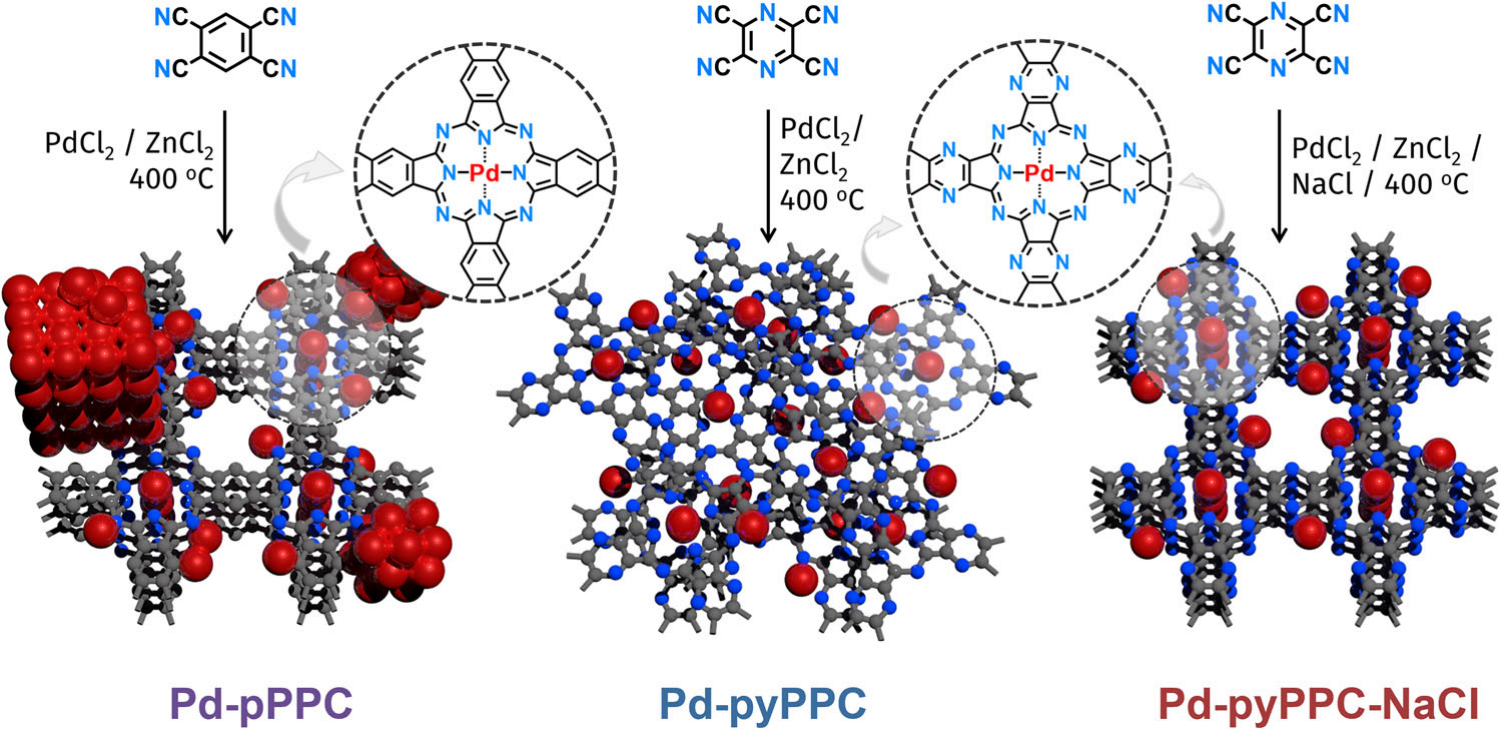
Schematic illustration of the design and preparation of porous polyphthalocyanines. The synthesis of COFs was achieved starting from 1,2,4,5‐tetracyanobenzene (Pd‐pPPC) and tetracyanopyrazine (Pd‐pyPPC) under mixed metal ionothermal conditions using the salt mixtures of PdCl_2_/ZnCl_2_ and PdCl_2_/ZnCl_2_/NaCl at 400 °C. Red spheres represent Pd atoms.

Herein, we report high metal loading SACs based on palladium porous polyphthalocyanine (Pd‐PPC) COFs (Figure 1), which are synthesized under mixed metal ionothermal conditions using metal salt mixtures of PdCl_2_/ZnCl_2_ or PdCl_2_/ZnCl_2_/NaCl starting from tetracyanobenzene (Pd‐pPPC) and tetracyanopyrazine (Pd‐pyPPC) by effectively combining the crystalline polymeric host formation with metal impregnation. We demonstrate the critical role of the pore chemistry of COF to mitigate the formation of Pd nanoparticles (NPs) and metal leaching through dynamic confinement as revealed by combined theoretical and experimental approaches. This structural control allows the incorporation of ultra‐high‐density dynamically confined metal ions with contents reaching up to 22.2 wt%, which is directly associated with their superior catalytic performance in the Suzuki–Miyaura cross‐coupling reaction under batch and continuous flow conditions.

## Results and Discussion

The design strategy is defined by considering the metal‐to‐ligand affinity as the principle to identify likely compositions. To assess the relative affinity of different ligands for metal ions, Ahrland and coworkers categorized metal ions into three classes such as class A, B, and borderline metals.^[^ ^23^ ^]^ Class A metals are composed of alkali metals and alkaline earth metals, whereas second and third‐row transition metals, such as palladium (Pd), platinum (Pt), rhodium (Rh), and ruthenium (Ru), are classified as Class B metals. In both class A and class B metals, the ligand plays a critical role in the complex stability. With respect to borderline metals, particularly first transition metal ions including Mn^2+^, Fe^2+^, Co^2+^, Ni^2+^, Cu^2+^, and Zn^2+^_,_ their complex stabilities follow the order given by the Irving–Williams series, which correlates nicely with the ionic radius and the charge density of the metal ions.^[^ ^24^ ^]^ Considering the high stability of Pd^2+^ on various N‐rich supports, we reasoned that it might indeed be possible to combine class B metals with the borderline ones in the mixed‐metal ionothermal synthesis,^[^ ^25^ ^]^ that is, the mixture of PdCl_2_ and ZnCl_2_, to form Pd‐based heterogeneous SACs. While molten ZnCl_2_ acts both as a catalyst and solvent and is used in significant excess relative to PdCl_2_, higher complex stability of Pd^2+^ relative to Zn^2+^ enables the incorporation of Pd into the COF support. Our simulations (Table S1) corroborate this behavior, that Pd remains strongly stabilized within the COF. Accordingly, the synthesis of Pd‐PPCs was performed under mixed‐metal ionothermal conditions using the metal salt mixture of PdCl_2_/ZnCl_2_ at 400 ^°^C for 24 h, starting from 1,2,4,5‐tetracyanobenzene, Pd‐pPPC, and 1,2,4,5‐tetracyanopyrazine, Pd‐pyPPC. The comparative analysis of these two polymers allowed us to probe the impact of nitrogen atoms to mitigate the formation of Pd‐NPs. We also explored the synthesis of the Pd‐pyPPC in the eutectic salt mixture of PdCl_2_/ZnCl_2_/NaCl at 400 ^°^C for 4,^[^ ^26^ ^]^ 12, and 24 h to form Pd‐pyPPC‐NaCl, as it was previously shown to improve the crystallinity of the resulting polymers.^[^ ^27^ ^]^ Powder X‐ray diffraction analysis revealed that a 24 h reaction time is required for achieving the high crystallinity and mitigating the formation of Pd NPs in Pd‐pyPPC‐NaCl (Figure S1).

**Figure 2:**
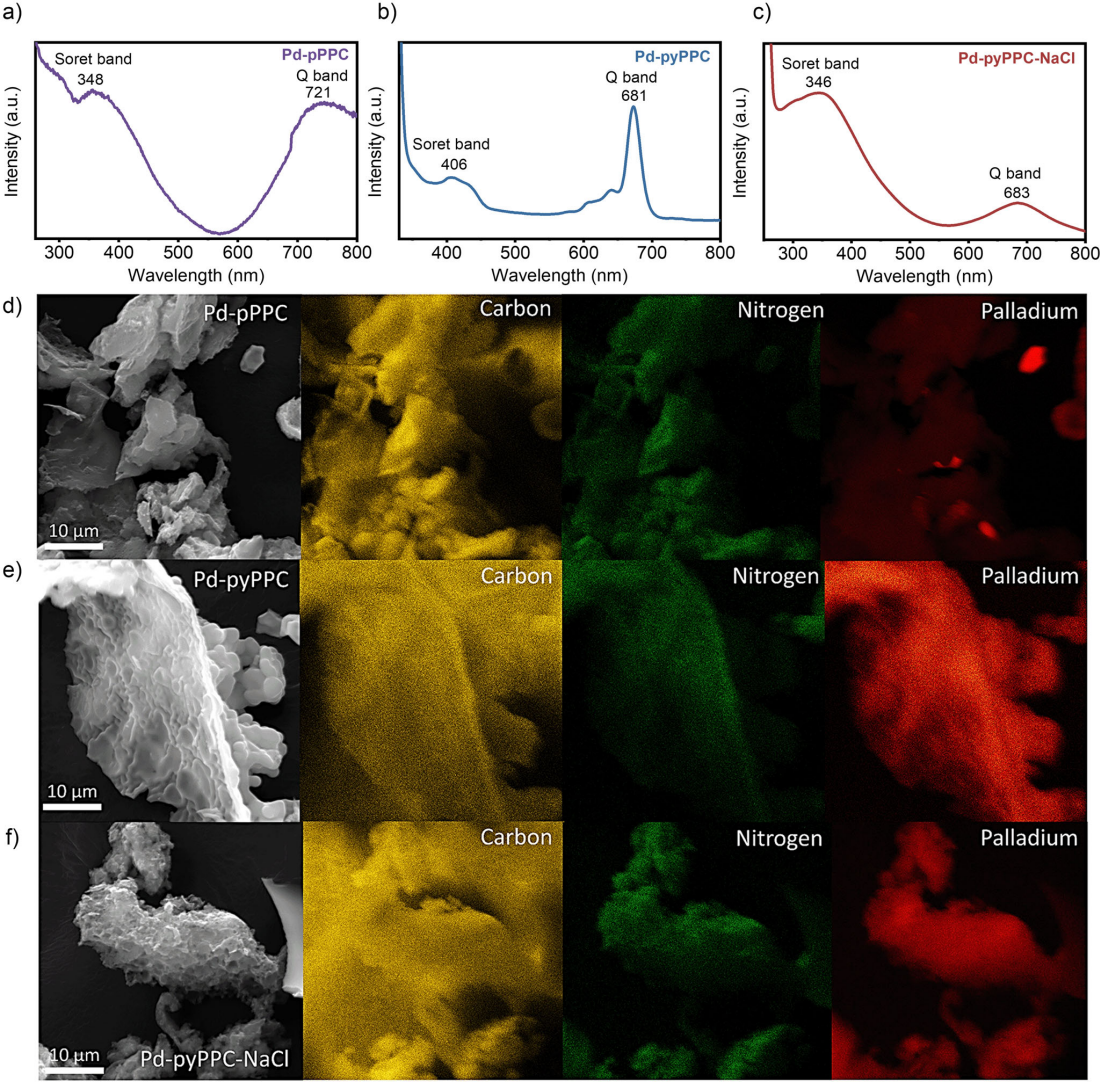
Structural and elemental characterization of Pd‐based porous polyphthalocyanine COFs. a)–c) UV–vis spectra of a) Pd‐pPPC, b) Pd‐pyPPC, and c) Pd‐pyPPC‐NaCl. d)–f) SEM images and EDX elemental mapping of d) Pd‐pPPC, e) Pd‐pyPPC, and f) Pd‐pyPPC‐NaCl. Homogeneous Pd ion distribution is evident in Pd‐pyPPC and Pd‐pyPPC‐NaCl, whereas Pd nanoparticle (NP) formation is observed clearly in Pd‐pPPC.

Ultraviolet‐visible (UV–vis) spectral analysis was performed to investigate the formation of phthalocyanine (PCs) moieties (Figure 2a–c). PCs display two characteristic absorption bands, namely, the *Q* band and the Soret band. The *Q* band, positioned at approximately 600–700 nm, corresponds to the degenerate electronic transition from the ground state (S_0_) to the first excited state (S_1_). The Soret band, appearing around 300–350 nm, on the other hand, arises from the electronic transition from the ground state (S_0_) to the second excited state (S_2_). All UV–vis spectra exhibit these characteristic absorption bands (Figure 2a–c). *Q* band broadenings in the case of Pd‐pPPC and Pd‐pyPPC‐NaCl were attributed to stronger *π–π* stacking interactions between the layers arising from their crystallinity.^[^ ^28^, ^29^ ^]^

**Figure 3:**
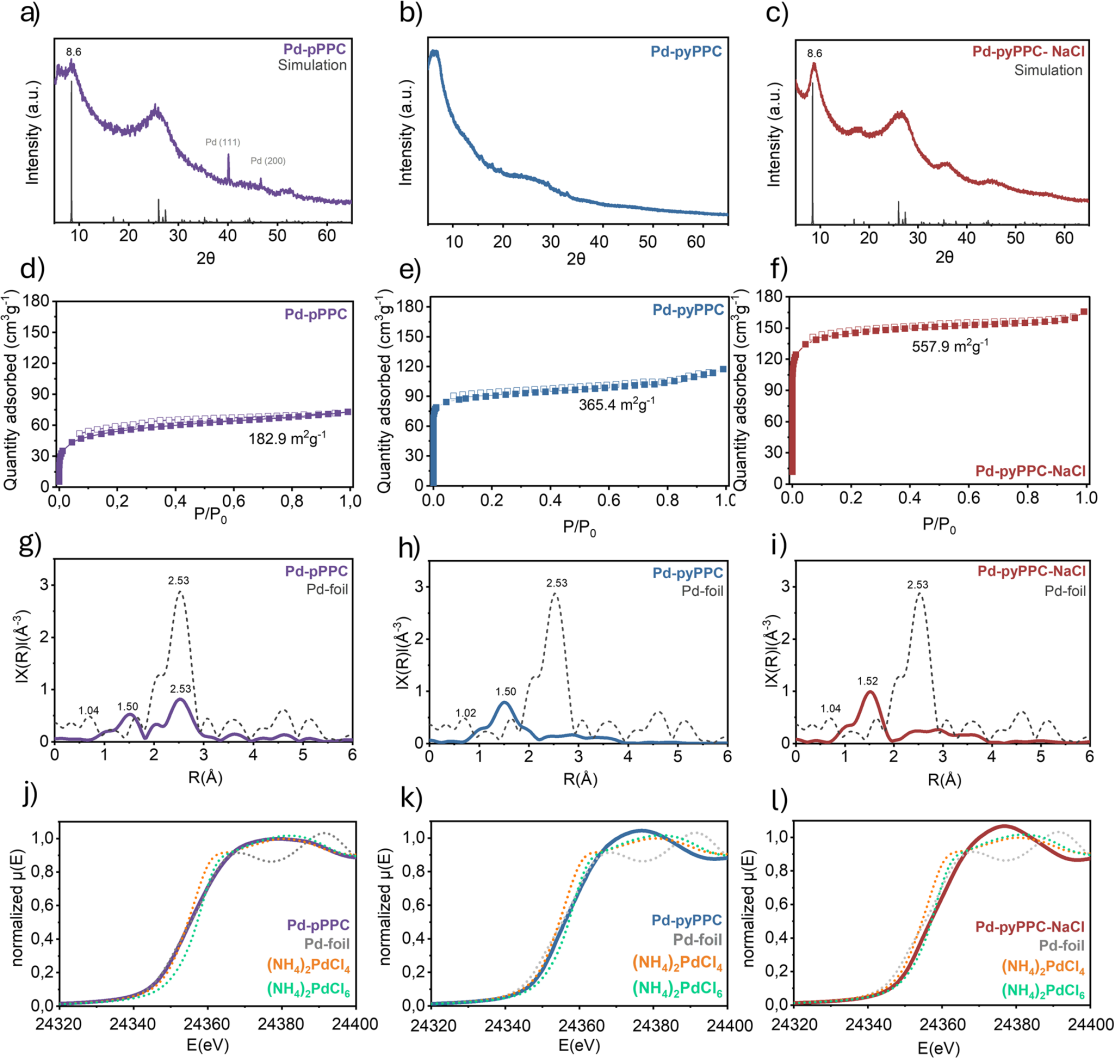
Structural and surface characterization of Pd‐based porous polyphthalocyanines. a)–c) Powder X‐ray diffraction (PXRD) patterns of a) Pd‐pPPC, b) Pd‐pyPPC, and c) Pd‐pyPPC‐NaCl, overlaid with simulated PXRDs. d)–f) N_2_ adsorption–desorption isotherms at 77 K for d) Pd‐pPPC, e) Pd‐pyPPC, and f) Pd‐pyPPC‐NaCl, showing corresponding Brunauer–Emmett–Teller (BET) surface areas. Filled and empty symbols indicate adsorption and desorption branches, respectively. g)–l) Normalized *k^2^*‐weighted Fourier transform (FT) magnitudes of the EXAFS (extended X‐ray absorption fine structure) spectra in radial distance (R) for (g) and (j) Pd‐pPPC, (h) and k) Pd‐pyPPC, and (i) and (l) Pd‐pyPPC‐NaCl.

The formation of PC moieties was also verified by Fourier transform infrared spectroscopy (FT‐IR) analysis, as presented in Figure S2. We observed the complete disappearance of nitrile (─C≡N) stretching band at around 2250 cm^−1^ accompanied by the appearance of the characteristic C─N stretching bands of pyrrole rings in the PCs at 1472 and 1290 cm^−1^, along with the phthalocyanine–metal coordination bond (M–N) at 893.5 cm^−1^.^[^ ^30^ ^]^ In the case of Pd‐pPPC and Pd‐pyPPC‐NaCl, C─N stretching bands of pyrazine units were also observed at 1373 and 1170 cm^−1^. Inductively coupled plasma‐optical emission spectrometry (ICP‐OES) analysis was performed to quantify the Pd contents of Pd‐PPCs (Table 1), which showed Pd contents of 19.2, 22.2, and 18.3 wt% for Pd‐pPPC, Pd‐pyPPC, and Pd‐pyPPC‐NaCl, respectively. Elemental analysis (EA) results (Table S2) further corroborated these findings. Pd‐pyPPC and Pd‐pyPPC‐NaCl showed significantly higher nitrogen contents of 27.7 and 28.6 wt%, respectively, compared to that of Pd‐pPPC, 18.3 wt%. In line with our earlier findings on the template effect under ionothermal conditions,^[^ ^31^ ^]^ that is, the stabilization of heteroatoms with metal ions, Pd^2+^ in this particular case, appears to mitigate the loss of heteroatom content, which is commonly observed for the polymers synthesized under ionothermal conditions. Thermogravimetric analysis (TGA) of Pd‐PPCs revealed (Figure S3) about 10 wt% mass loss below 200 °C, originating from the loss of adsorbed/coordinated water molecules as well as residual solvent molecules. Whereas Pd‐pPPC and Pd‐pyPPC‐NaCl were found to be stable up to 500 °C under air, Pd‐pyPPC showed relatively lower thermal stability. All the polymers showed a residual mass in the range of 17–28 wt% arising from their high Pd contents. To probe the morphology of Pd‐PPCs, scanning electron microscopy (SEM) analysis was performed (Figures 2d–f and S4). We observed micron‐sized particles with film‐like morphology in the case of Pd‐pPPC and Pd‐pyPPC. Moreover, we could also observe the micrometer‐sized cavities in the Pd‐pyPPC‐NaCl, suggesting the templating effect of NaCl.^[^ ^31^, ^32^ ^]^ In addition, scanning electron microscopy‐energy dispersive X‐ray (SEM‐EDX) analysis (Figure 2d–f) revealed the uniformly distributed Pd atoms in the Pd‐pyPPCs. Whereas Pd‐PPC exhibited the formation of Pd nanoparticles, Pd‐pyPPC showed the highest Pd intensity among the samples analyzed, in agreement with the ICP‐OES data. To probe the crystallinity of Pd‐PPCs, powder X‐ray diffraction (PXRD) analysis was conducted (Figure 3a–c and Tables S2 and 3). Pd‐pPPC features (Figure 3a) peaks at 2θ = 8.6 and 25.5°, which can be indexed to (100) and (001) planes, respectively, supporting the formation of a 2D COF structure.

**Table 1:** BET surface area analysis of Pd‐PPCs along with their Pd loadings.

	BET^a)^ (m^2^ g^−1^)	*S*_micro_ ^b)^ (m^2^ g^−1^)	*S*_ext_ (m^2^ g^−1^)	*V*_total_ ^c)^ (cm^3^ g^−1^)	*V*_micro_ ^d)^ (cm^3^ g^−1^)	*V*_external_ ^e)^ (cm^3^ g^−1^)	Pd^f)^ (wt%)
Pd‐pPPC	182.9	172	10.9	0.095	0.078	0.017	19.2 ± 0.32
Pd‐pyPPC	365.4	269	56.2	0.201	0.115	0.086	22.2 ± 0.28
Pd‐pyPPC‐NaCl	557.9	391	137	0.245	0.163	0.082	18.2 ± 0.33

^a)^ Brunauer − Emmett − Teller (BET) surface area calculated over the pressure range (*P*/*P*_0_) of 0.01 − 0.11.

^b)^ Micropore surface calculated using the *t*‐plot method.

^c)^ Total pore volume obtained at *P*/*P*_0_ = 0.99.

^d)^ Micro pore volume calculated using the *t*‐plot method.

^e)^ *V*_external _= *V*_total_–*V*_micro_.

^f)^ Metal content is calculated by Inductively coupled plasma‐optical emission spectrometry (ICP‐OES).

However, we also observed characteristic diffraction peaks at 2θ = 40° and 47°, corresponding to the (111) and (200) planes of face‐centered cubic (fcc) palladium, respectively. This result indicates that Pd‐pPPC cannot suppress the Pd crystal growth. Pd‐pyPPC diffractogram, on the other hand, showed no palladium crystal formation, and a loss of polymer crystallinity (Figure 3b). The use of a eutectic salt mixture of ZnCl_2_/NaCl significantly improved (Figure 3c) the crystallinity of Pd‐pyPPC‐NaCl while retaining high Pd content without Pd crystal formation, owing to the high nitrogen content of the polymers. Brunauer–Emmett–Teller (BET) surface area analysis was conducted using N_2_ at 77 K (Figures 3d–f and S5) to evaluate the porosity of Pd‐PPCs. The surface areas of Pd‐pPPC, Pd‐pyPPC, and Pd‐pyPPC‐NaCl were determined to be 182.9, 365.4, and 557.9 m^2^ g^−1^, respectively (Table 1). Pd‐pPPC, Pd‐pyPPC, and Pd‐pyPPC‐NaCl displayed type I isotherms, indicating the formation of a microporous network. To determine the pore size and pore size distribution of the polymers, the non‐local density functional theory (NLDFT)^[^ ^33^ ^]^ (Carbon–N_2_ at 77 K, 2D‐NLDFT heterogeneous surface model) was applied, which showed two distinct pores in the range of ultramicropore, between 0.46–0.54 nm, and micropore, between 0.91–1.16 nm (Figure S6).

We further probed (Figure S7) the molecular connectivity of Pd‐pyPPC and Pd‐pyPPC‐NaCl by X‐ray photoelectron spectroscopy (XPS) analysis. The deconvoluted N 1s spectra (Figures S7E and S7F) revealed the characteristic N1 (Aza bridging) and N2 (Pyrrolic N) at 398.4 and 397.1 eV for Pd‐pyPPC and at 398.3 and 397.8 eV for Pd‐pyPPC‐NaCl, respectively, which are well matched with the previously reported metallophthalocyanines.^[^ ^21^, ^34^ ^]^ The Pd 3d spectra (Figures S7G and S7H) revealed the presence of Pd^2+^ in both polymers. The corresponding Pd 3d_3/2_ and Pd 3d_5/2_ peaks were observed at 342.1 and 336.8 eV for Pd‐pyPPC and at 342.7 and 337.5 eV for Pd‐pyPPC‐NaCl, respectively. Compared to the previously reported Pd‐phthalocyanines, the peak positions slightly shifted to lower binding energy, presumably due to differences in the electronic structure arising from extended conjugation and interlayer stacking. Moreover, these binding energies do not match those of PdCl_2_, PdO, and Pd(0), 342.8 eV (Pd 3d_3/2_) and 337.9 eV (Pd 3d_5/2_) of PdCl_2_,^[^ ^35^ ^]^ 340.9 eV (Pd 3d_3/2_) and 335.7 eV (Pd 3d_5/2_) of Pd(0),^[^ ^35^ ^]^ 342.5 eV (Pd 3d_3/2_) and 337.2 eV (Pd 3d_5/2_) of Pd(0).

We also conducted X‐ray absorption structure (XAS) analysis of Pd‐PPCs (Figure 3g–l). The observation of Pd–Pd peak in Pd‐pPPC corroborates the presence of Pd nanoparticles (Figure 3g). Pd‐pPPC showed the coexistence of Pd^2+^ and Pd^0^ species (Figure 3j), in agreement with the PXRD analysis. Pd‐pyPPC (Figure 3h,k) and Pd‐pyPPC‐NaCl (Figure 3i,l) plots, on the other hand, are close to the (NH_4_)_2_PdCl_4_ line and do not match with (NH_4_)_2_PdCl_6_. This observation suggests that the predominant oxidation state of Pd is + 2, with some higher oxidation states present in Pd‐pyPPC‐NaCl but not exceeding + 4. The peak 1.50 Å in the R‐space spectra of Pd‐PPCs (Figure 3h,i), which can be assigned to the palladium coordination to nitrogen or carbon.^[^ ^36^ ^]^ In addition, the lack of Pd–Pd peaks in R‐space data of Pd‐pyPPCs points toward isolated Pd atom formation (Figure 3h,i).

**Figure 4:**
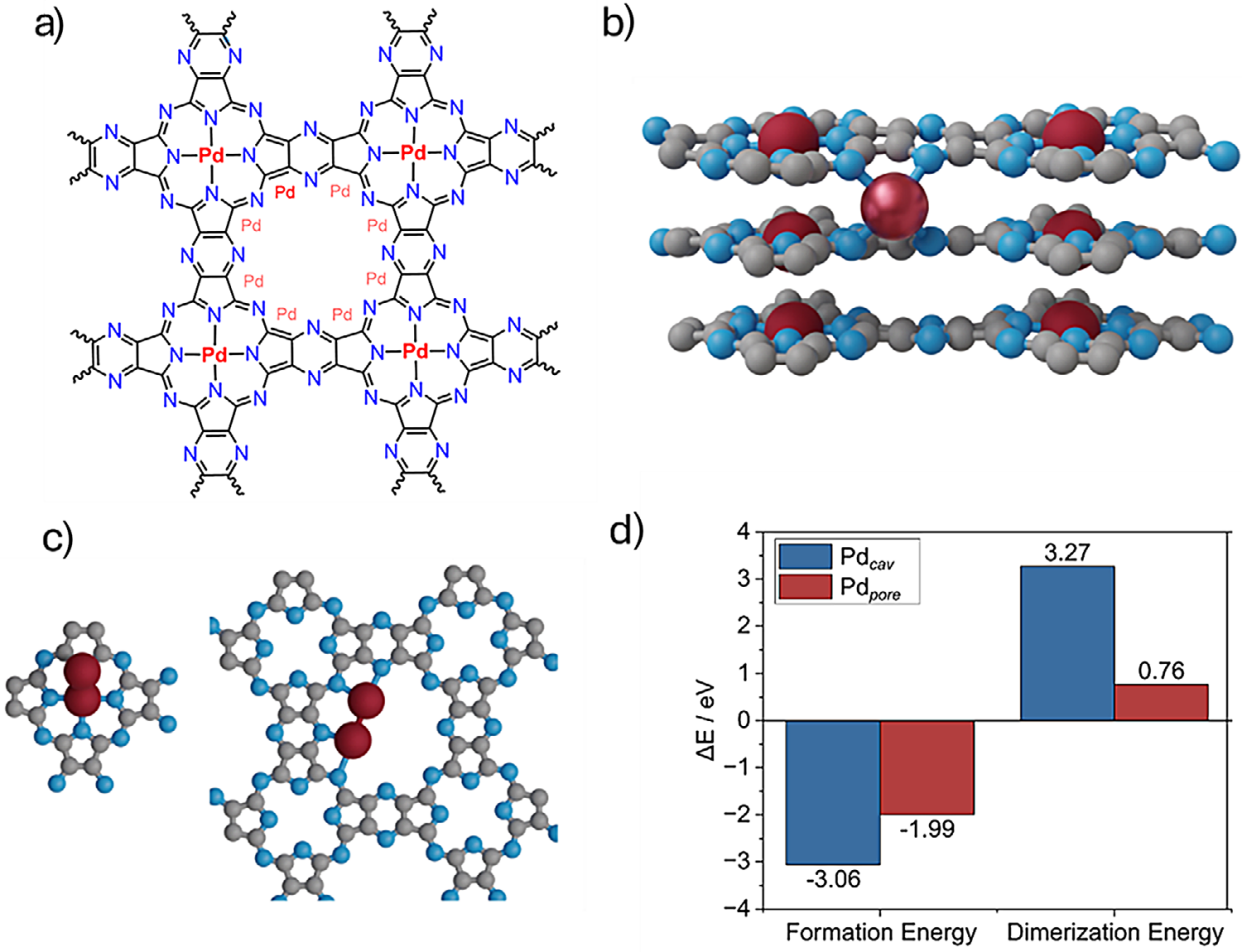
Structural model of Pd‐pyPPC COFs. a) Top view of the Pd‐pyPPC COF showing Pd atoms embedded in distinct coordination environments. Graphitic nitrogen atoms are shown in dark blue, and the ones in pore are medium blue. Pd*_cav_* is shown in dark red and Pd*_pore_* in red. Transparent red highlights all available coordination sites for Pd within the pore. b) Front view of a three‐layered COF structure illustrating the anchoring of Pd*_pore_*. c) Structural representation of Pd*_cav_* (left) and Pd*_pore_* (right) dimers. d) Formation energy of Pd species relative to the COF with two adsorbed hydrogen atoms (*2H*) and PdCl_2_. The plot also presents dimerization energies referenced to adsorbed Pd (*pore* or *cav*) and bulk Pd (Pd*_bulk_*).

To further understand the nature of Pd binding sites in the COF structure, we performed density functional theory (DFT) (PBE + D3) simulations. The native Pd‐pyPPC‐NaCl COF was built as a two‐dimensional layered polymer with a square phthalocyanine pocket, labelled cav, and forms a periodic network with large internal pores of 7.6–8.6 Å (radii from N–N). Each pore is delimited by eight nitrogen atoms of the framework, Figure 4a. These two‐dimensional layers stack along the *z*‐direction, and their cohesion is governed by non‐covalent interactions such as *π–π* stacking and van der Waals forces. Among the possible stacking modes, a nearly eclipsed AA configuration is identified to be the most stable, based on the thermodynamic data presented in Table S5 and Figure S9. This configuration maintains the accessible porosity key for optimal catalytic and sorption performance for flow reactions. Within this structural motif, Pd atoms may be anchored in three distinct types of binding sites, to the phthalocyanine cavity, Pd_cav_, adopting the typical square–planar coordination environment of Pd(II) complexes. It can also anchor at the corners of the internal pores, Pd_pore_, where Pd binds to two nitrogen atoms from the framework as well as to the nitrogen atoms located between two different layers. The interlayer spacing along the *z*‐axis is consistently around 3.6 Å for both pristine COF and its Pd derivatives. This spacing is characteristic of the stacking of 2D polymer held by non‐covalent interactions since the perturbation of Pd is only local and does not affect the average d‐spacing. As shown in Table S6, the interactions remain stable up to the highest experimentally relevant metal loading. The number of cav and pore positions per gram of host is 1.16·10^22^ sites·g^−1^ of Pd‐pyPPC. The insertion of Pd in the hydrogenated cavity with respect to PdCl_2_, is −3.06 eV (with the concomitant formation of two 2 HCl molecules) and in the pore, −1.99 eV, retaining the Cl atoms in a square planar configuration. Elimination of the two Cl atoms in the pore configuration requires more than 2 eV. The agglomeration of the Pd atoms was also studied in this system. The dimerization at the cavity is calculated (Figure 4c) to be endothermic by 3.27 eV with respect to Pd_cav_ and one additional Pd_bulk_ atom as the reference state. In the pore, Figure 4c, it is also found to be endothermic, 0.76 eV with respect to Pd_pore_ and one additional Pd_bulk_ as a reference. In addition, it is important to note that the number of atoms on the Pd‐pyPPC remains one order of magnitude lower than the total number of available cav + pore sites. This disparity plays a significant role in the configurational entropy of the system, *S* = kB·ln Ω, Ω, is the combinatorial of Pd and sites, leading to the number of possible configurations. For the 22.2 wt% loading case, the configurational contribution to Gibbs free energy of the system is approximately 0.18 eV/Pd at 673 K, at which this SAC is formed. The entropy gain associated with dispersing Pd atoms across a much larger number of available sites favors atomic isolation and homogeneity over agglomeration. Another contribution to the dispersion is the mobility of these atoms. Pd_pore_ atoms are highly mobile, jumping between different N‐configurations of the same pore, Figure 4b, which requires a barrier of 0.1 eV, corresponding to an approximate frequency of 10^12^ events per second, while jumping from one pore to the next along the *z*‐direction requires around 1.0 eV, occurring at much slower rate of 1.25·10^−3^ Hz at room temperature. Therefore, the Pd atoms in the pore can be seen as confined isolated species, nesting in only one two‐dimensional pore, dynamically moving along the eight equilibrium positions. These results shed light on the viability of the other two materials. For the Pd‐pPPC, the N content in the pore is far too small and does not allow for the PdCl_2_ to interact and form a square–planar structure. Consequently, the pores do not contain anchoring sites for Pd to stay, and nanoparticles are formed. In the second case, the disordered polymer contains the anchoring site, but the lack of crystallinity will limit confinement. Taken together, these observations indicate a low propensity for Pd clustering in this system, in agreement with the EXAFS analysis.

The presence of uniformly distributed high‐density single Pd atom sites in the Pd‐pyPPC and Pd‐pyPPC‐NaCl and differences in their porosity prompted us to evaluate their catalytic performance as heterogeneous SACs in the Suzuki–Miyaura cross‐coupling reaction.^[^ ^36^, ^37^, ^38^ ^]^ Initially, we conducted screening reactions using Pd‐pyPPC (Table S7) to determine the most suitable base. In terms of the reaction yield, we observed the following trend: K_2_CO_3 _> Cs_2_CO_3 _> NaOH > KOH > Na_2_CO_3 _> TEA, thus we conducted all the reactions using K_2_CO_3_ as the base. The superior performance of K_2_CO_3_ in the reaction can be attributed to its high solubility both in organic solvents and water, which facilitates better dispersion and interaction with substrates and the catalyst. Additionally, the balanced nucleophilicity of carbonate ions from K_2_CO_3_ enhances the stability of catalytic intermediates and minimizes side reactions, improving selectivity.^[^ ^39^ ^]^ Accordingly, we performed (Table S8) the initial catalytic tests using aryl halides with electron‐withdrawing (EWG) or electron‐donating groups (EDG) under batch conditions. We observed consistently higher conversion yields and selectivity for aryl halides with an EWG, that is 4‐cyanobromobenzene. Whereas Pd‐pPPC and Pd‐pyPPC‐NaCl showed yields of 50% and 83%, respectively, Pd‐pyPPC showed higher yields of 89% (Table S8), which is a highly competitive performance compared to the homogeneous catalysts under the same conditions. We also tested two commonly used commercial catalysts, palladium on carbon Pd/C (5 wt%) and tetrakis(triphenylphosphine)palladium(0) Pd(PPh_3_)_4_, under identical reaction conditions for comparison. Both catalysts performed well, with Pd/C achieving an 85% yield and Pd(PPh_3_)_4_ reaching 87% yield, which are comparable to the yields achieved by Pd‐pyPPC and Pd‐pyPPC‐NaCl systems. In the case of substrates bearing EDGs such as 4‐bromotoluene and 4‐bromoanisole, however, we observed moderate conversion yields (Table S8). This is not surprising as the EWGs facilitate the oxidative addition step in the Suzuki–Miyaura reaction, whereas EDG hinders it. Noticeably, we observed (Figure 6b,c) the highest yields for the Pd‐pyPPC and Pd‐pyPPC‐NaCl, whereas Pd‐pPPC showed lower yields for all the aryl halide substrates. We also synthesized a Pd–PC it model compound^[^ ^40^ ^]^ and tested it under identical batch and flow conditions, which showed considerably lower yields of 24.4% (decreased to 5.9% after reuse) and 9.4%, respectively (Figure S9).

**Figure 5:**
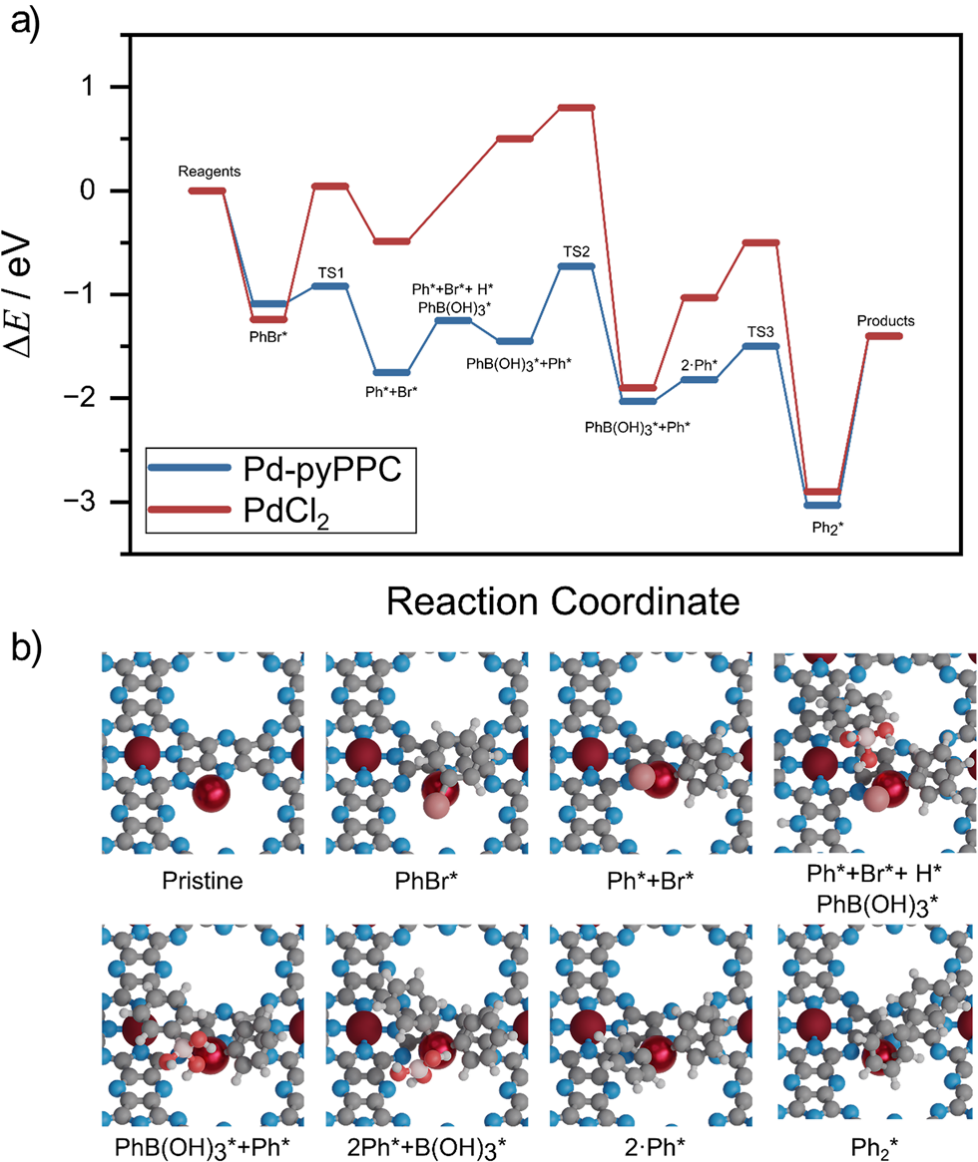
a) Thermodynamic comparison between Pd_pore_‐pyPPC, Pd‐pPPC, and Pd_cav_‐pyPPC, blue, red, and yellow, respectively, for the Suzuki–Miyaura cross‐coupling reaction. b) Representation of the reaction models for the reaction within the COF. Color code: O: red, B: light brown, C: gray, N: light blue, H: light gray, Pd*_cav_*: dark red, Pd*_pore_*: metallic red.

The mechanism of the Suzuki–Miyaura cross‐coupling reaction was analyzed via DFT. To account for the high mobility of Pd atoms in the pore, we put particular attention to the ongoing debate of the homogeneous versus heterogeneous nature of active species.^[^ ^36^, ^41^, ^42^ ^]^ The reaction is shown in Figures 5, and S9–S12. To gain insight into the electronic environment of the catalytic center, core‐level binding energy shifts on each step of the reaction have been calculated (∆BE). Both energies and a ∆BE have been collected in Table S10. The reaction was studied on two different scenarios, the first one is if the process occurs in the Pd_pore_, and the second is considering the case it has been released to the media. In both scenarios, Pd leaching is prevented due to its interaction with the large number of anchoring sites in the pore. The first step is the adsorption and a former oxidative addition of PhBr to Pd_pore_, a process that goes through a transition state −0.92 eV, leading to C─Br bond cleavage. This intermediate has a ∆BE = +2.4 eV on the Pd atom, showing the oxidative addition. As can be seen, the interaction of Pd with the COF support lowers the energies since it stabilizes the intermediates more than PdCl_2_. Prior to transmetalation, the activated phenyl borate absorbs, positioning H + within the pore while the borate interacts with the Pd_pore_ atom (−1.25 eV, ∆BE = +2.3 eV).

As the borate bonds to Pd, it leads to the release of HBr and to the barrierless cleavage of the Ph─B(OH)_3_ bond. This results in both fragments—B(OH)_3_* and Ph*—coordinated to Pd (−1.45 eV, ∆BE = +2.6 eV). The reaction concludes with reductive elimination, transitioning from Pd‐bound Ph groups (−1.82 eV, ∆BE = +2.6 eV) to the final product (−3.03 eV, ∆BE = +0.46 eV) via a low‐energy transition state. The product is released into the gas phase, restoring the catalyst with a final energy of −1.40 eV. Besides, the differences in energy between the two different Pd, both can be achieved under reaction conditions. Although studies on heterogeneous SACs containing Pd^2+^ are limited,^[^ ^36^, ^43^ ^]^ Pd(II)/Pd(IV) cycle mechanism has been proposed in the cross‐coupling reactions for both homogeneous^[^ ^44^, ^45^ ^]^ and heterogeneous catalysts using supports such as g‐C_3_N_4_.^[^ ^36^, ^45^, ^46^ ^]^ The core‐level electron shift was obtained for the intermediates, revealing a trend similar to the experimental observation with values larger than 2 eV. Accordingly, we reasoned that the Suzuki–Miyaura cross‐coupling reaction using Pd‐pyPPCs can go through Pd(II)/Pd(IV) cycle owing to the availability of additional nitrogen binding sites within its pore, which can effectively stabilize the intermediates through the reaction pathway. We also benchmarked the catalyst against Pd‐pPPC, and the observed trends are consistent with experimental results. The inferior performance of Pd‐pPPC is mainly related to Pd leaching and subsequent nanoparticle formation. Figures S8–10 show clearly that the difference between the two is due to extra nitrogen atoms in the pore, which is crucial for Pd anchoring.

Recycling experiments of Pd‐pyPPC and Pd‐pyPPC‐NaCl showed a negligible decrease in their catalytic performance over five reuse cycles, in stark contrast with the drastic decrease in catalytic activity observed for the homogeneous catalysts.^[^ ^38^ ^]^ These findings indicate clearly that the high‐nitrogen contents of Pd‐pyPPC and Pd‐pyPPC‐NaCl can effectively stabilize Pd^2+^, thus leading to exceptional recyclability. Critically, the PXRD analysis of recycled samples revealed that both Pd‐pPPC and Pd‐pyPPC‐NaCl maintained their crystallinity (Figure S13). In addition, we did not observe any palladium crystal growth in both Pd‐pyPPC and Pd‐pyPPC‐NaCl. The emergence of broad features in the case of Pd‐pyPPC suggests the restructuring of the polymer network following the catalytic tests.

**Figure 6:**
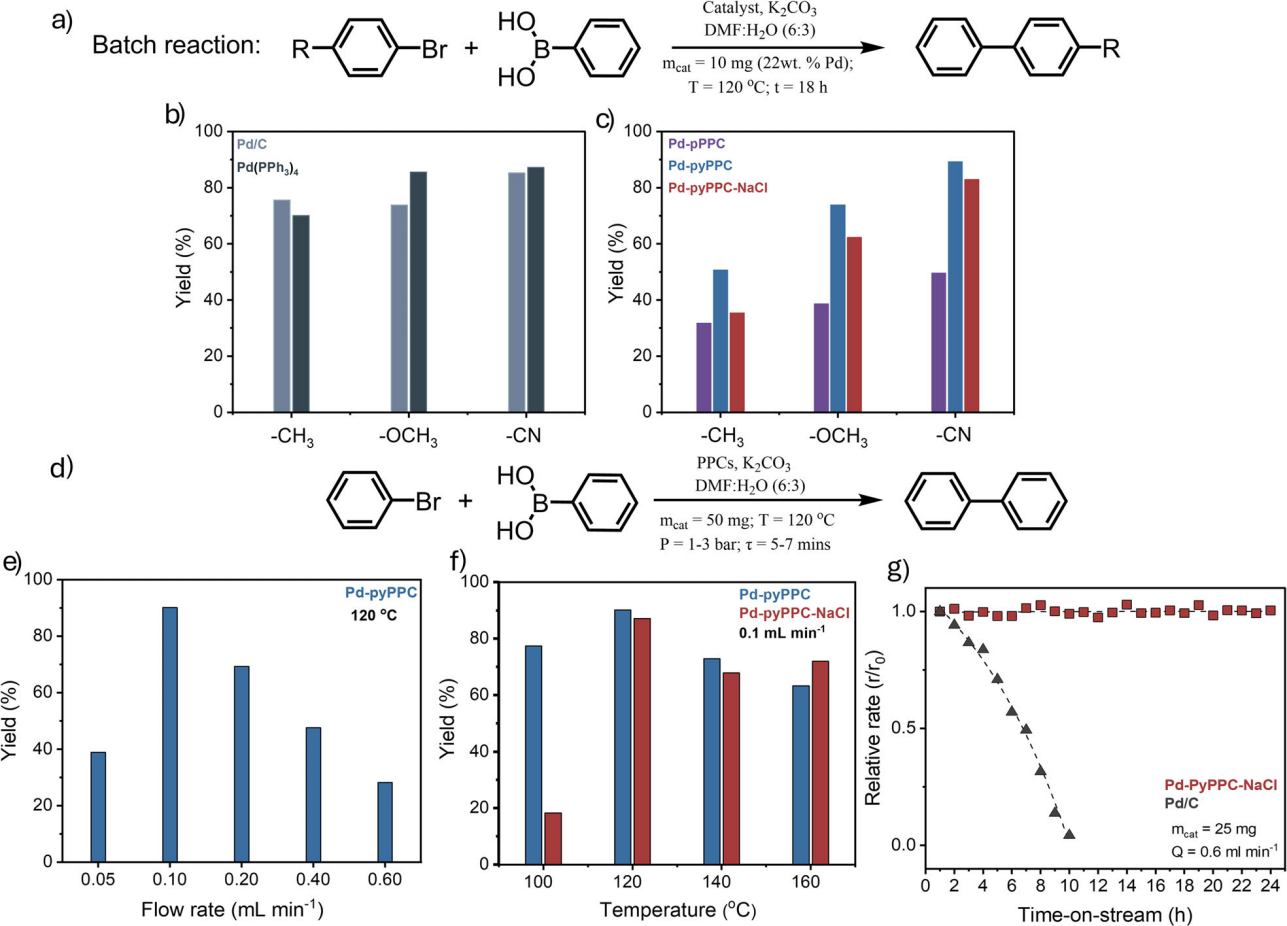
Performance comparison of Pd–PPC catalysts and commercial benchmarks in Suzuki–Miyaura cross‐coupling under batch and continuous‐flow conditions. a), Schematic representation of the Suzuki–Miyaura cross‐coupling between three aryl bromide substrates and phenyl boronic acid in a batch reaction using commercial catalysts (Pd/C, Pd(PPh_3_)_4_, and Pd‐PPCs. b) and c, Batch reaction yields for three aryl bromide substrates using commercial catalysts (Pd/C, Pd(PPh_3_)_4_) and Pd–PPCs. d), Schematic representation of the Suzuki–Miyaura cross‐coupling between bromobenzene and phenyl boronic acid in a continuous‐flow setup employing Pd‐pyPPC and Pd‐pyPPC‐NaCl. e), Flowrate dependence study of Pd‐pyPPC. f), Temperature dependency study of Pd‐pyPPC and Pd‐pyPPC‐NaCl. g), Relative rate (*r/r_0_*) as a function of on‐stream duration for Pd‐pyPPC‐NaCl compared with Pd/C under low‐conversion conditions. Yields are determined using GC‐MS.

We also performed leaching tests by measuring Pd content after hot filtration with water or DMF using ICP‐OES. We observed significant Pd leaching from Pd‐pPPC, which can be attributed to the weak interactions between the confined palladium crystals and the surface of Pd‐pPPC. Moreover, the hot‐filtrated solution of Pd‐pPPC exhibited a dark‐greenish color, while the solutions of Pd‐pyPPC and Pd‐pyPPC‐NaCl were transparent. Both Pd‐pyPPC and Pd‐pyPPC‐NaCl did not show any leaching within the detection limit of the ICP‐OES instrument. All these observations agree with the stabilization of Pd atoms through dynamic confinement in the COF. For the Pd‐pPPC, once the Pd is leached out of the host, there are not sufficiently strong anchoring points for the Pd to redisperse in the material. Alternatively, for the polymeric and crystalline forms of Pd‐pyPPC, under the Suzuki–Miyaura cross‐coupling reaction conditions, Pd atoms remain confined as in the diffusion path of Pd toward the solution, the atoms will find enough anchoring sites (0.01 mol g^−1^ host) to retain the dynamic N bonds and prevent the leaching. Thus, the configurational entropy of the system due to the massive number of anchoring points compared to the number of particles is ultimately responsible for their behavior as Pd single atom sponges. This behavior aligns with the volcano plot of flow rate (Figure 6e): if the flow is too low, reactants will not effectively interact with the catalyst, while if the flow is too high, the species do not have enough time interacting with the catalysts, thus lowering the reaction rates.

We also characterized (Figure S14) Pd‐PPCs before and after the Suzuki–Miyaura cross‐coupling reaction (in a batch) using EXAFS. Pd‐pPPC showed a change in the oxidation state of Pd from 0 to 2 + as evidenced by the line shifts to the higher absorption energy in XANES (Figure S14A), accompanied by the decrease in the amplitude of Pd–Pd distance in the R‐space analysis (Figure S14B), which is because Pd crystals detach from Pd‐pPPC into the solution during the reaction. The lines in XANES for Pd‐pyPPC and Pd‐pyPPC‐NaCl perfectly overlap with each other before and after the reaction (Figures S14C and S14D). Moreover, their R‐space spectrum is also fully overlapped (Figures S14E and S14F) and does not show Pd–Pd bonding, which supports that no Pd nanoparticle formation occurs, in agreement with the PXRD results. All XANES and R‐space analysis spectra of Pd‐pyPPC and Pd‐pyPPC‐NaCl support the presence of isolated Pd atoms after use. It is important to note that the properties measured by XANES and EXAFS are averaged for the full sample, meaning that the fact that these observables are the same does not imply that the atoms are sitting in exactly the same atomic positions. Therefore, the observations are compatible with a dynamic confinement of isolated Pd atoms.

We also performed (Figure 6) the continuous Suzuki–Miyaura coupling reactions using a flow reactor under optimized reaction conditions (for optimization details, see Table S11). We identified an optimal flow rate of 0.1 mL min^−1^ and a temperature of 120 °C, while maintaining a differential pressure of 1–2 bars. Under these conditions, Pd‐pyPPC‐NaCl exhibited a comparable yield to Pd‐pyPPC, achieving yields of 87% and 90%, respectively (Figure 6d,e), in contrast to batch reactions. This comparable performance could be attributed to the higher surface area of Pd‐pyPPC‐NaCl relative to Pd‐pyPPC, which allows for improved accessibility to active catalytic sites. This, in turn, enhances mass transfer and overall catalytic performance, particularly in continuous flow reactions.

The catalytic performance and stability of Pd‐PyPPC‐NaCl was further examined under continuous flow for a 24 h period under low conversion conditions at a flow rate of 0.6 mL min^−1^, which demonstrated notable stability with the relative rate (*r*/*r*_0_) near 1.0 throughout the experiment without significant deviations, whereas Pd/C (5 wt%) exhibited a lower yield and a significant stability loss over the same period despite having comparable performance under batch conditions (Figure 6g). Additionally, comparative leaching tests were performed for Pd‐pyPPC‐NaCl and commercial Pd/C before and after catalytic reactions. Pd‐pyPPC‐NaCl showed no Pd leaching after the reaction, with a residual Pd content of 18.2 wt% representing only a 0.1 wt% difference, which falls within the experimental error. In contrast, Pd/C exhibited significant Pd loss post‐reaction, confirming the superior structural stability and metal retention of Pd‐pyPPC‐NaCl under operating conditions (Figure S15 and Table S12). This result demonstrates the robust performance of Pd‐pyPPC‐NaCl over time‐on‐stream, underscoring the catalyst's stability and self‐healing properties for industrial applications. The high catalytic activity Pd‐pyPPC‐NaCl under continuous‐flow conditions further verifies the potential of mixed‐metal ionothermal synthesis for the preparation of SACs and underscores their suitability as robust heterogeneous catalysts in industrial continuous‐flow processes.

## Conclusion

We demonstrated highly efficient synthesis of SACs based on Pd‐phthalocyanine COFs with ultra‐high metal loadings up to 22.2 wt% using mixed‐metal ionothermal synthesis. This synthetic approach effectively combines the formation of the crystalline polymeric host with a metal impregnation, thus introducing a new strategy for the synthesis of SACs with high metal loadings. Furthermore, the dynamic confinement of Pd SACs within the COF matrix enables a self‐healing strategy, increasing the robustness of the material under continuous flow conditions. Overall, our findings highlight the potential of this methodology for producing high‐performance heterogeneous SACs based on crystalline organic supports with tunable porosity and exceptional single‐site metal density and tunable surface areas for cross‐coupling reactions.

## Supporting Information

The authors have cited additional references within the Supporting Information.

## Author Contributions

K.S.S. and M.N. contributed equally to this work, conducted all experimental work, including COF synthesis, catalytic tests, data analysis, and drafted the manuscript. S.P. and M.N. performed X‐ray absorption spectroscopy analysis. T.A. supported the SEM imaging. P.W.F. contributed to the XPS measurements. J.M.G.A., A.R.F., and N.L. performed the DFT simulations and assisted with the manuscript writing. F.G. assisted with the COF structure elucidation. A.C. conceptualized and supervised the project and provided guidance on manuscript writing and revision. All authors contributed to scientific discussions and reviewed the manuscript.

## Conflict of Interests

The authors declare no conflict of interest.

## Supporting information



Supporting information

## Data Availability

The data support the findings of this study are available in Zenodo at https://doi.org/10.5281/zenodo.18090940. The computed structures can be found in https://doi.org/10.19061/iochem‐bd‐1‐377.
