# Supplementary material for: Dynamic Confinement Approach for High Metal Loading Single‐Atom Catalysts Based on Covalent Organic Frameworks
Source: Angew Chem Int Ed Engl. 2026 Jan 1;65(7):e22238. doi: 10.1002/anie.202522238 (PMC12887631; doi:10.1002/anie.202522238)
Supplement: Supplementary file 1 — Supporting information [file ANIE-65-e22238-s001.pdf]

Materials

All chemicals were purchased from Sigma Aldrich and used as received without further purification. Zinc (II) chloride (puriss., crystalline powder 98-100.5 %), palladium chloride (anhydrous, > 99.99 %, trace metals basis), sodium chloride (ACS reagent, > 99.0 %), 1,2,4,5-tetracyanobenzene (97 %). 1,2,4,5-tetracyanopyrazine is synthesized by following a previously reported procedure.[1]

Characterizations

The powder X-ray diffraction (PXRD) patterns of Pd-PPCs are collected on a STADIP micro area X-ray diffractometer using CuK_α (λ = 0.154 nm) radiation. PXRD spectra were recorded in the 2θ range of 2 – 60° or 70° with a 2θ step size of 1.0 and a step time of 30 s. The analysis of various chemical bonds and functional groups in each sample was performed by Fourier-transform infrared spectroscopy (FT-IR, Perkin Elmer). X-ray photoelectron spectroscopy (XPS) analysis was performed with a Sigma 2 X-ray photoelectron spectrometer (ThermoFisher Scientific) equipped with a non-monochromatic 200 W Al K_α source, a hemispherical analyzer, and a seven-channel electron multiplier. A pass energy of 50 eV and 25 eV is usually used for collecting the survey and the narrow scans, respectively. A step size of 1 eV and 0.1 eV is used for collecting the survey and narrow scans. Ultraviolet-visible (UV-Vis) spectroscopy analysis was carried out by using Lambda 900 (Perkin Elmer). Thermogravimetric analysis (TGA) was conducted under air and N_2 atmosphere from 25 to 1000 °C using the Mettler Toledo TGA/DSC 3+ instrument. The morphology of Pd-PPCs was conducted using a field emission scanning electron microscope (FE-SEM, Tescan Mira3) and Energy Dispersive X-Ray Analysis (EDX, Oxford Instruments). Metal content analysis was carried out by using Inductively Coupled Plasma Optical Emission Spectroscopy (ICP-OES, OPTIMA7000 DV). The textural parameters of Pd-PPCs were evaluated by using N_2 adsorption and desorption isotherms, which were measured at 77 K on a Micrometrics 3Flex Surface Characterization Analyzer. All samples were degassed at 100 °C for 16 h prior to the analysis. The specific surface areas of samples were calculated using the BET and Langmuir models in the pressure range where the term $V(1-P/P_o)$ continuously increases with P/P_o in the Rouquerol plot. Pore size distributions of samples were calculated from Ar isotherms according to the nonlocal density functional theory (NLDFT) method (model: Carbon-N_2-77K, 2D-NLDFT Heterogeneous Surface) via Saieus software. X-ray absorption spectroscopy (XAS) spectra of the Pd K-edge were collected at the SuperXAS beamline at Paul Scherrer Institute (PSI), which operated in top-up mode at 2.4 GeV and a ring current of 400 mA. A silicon-coated mirror (which also reduced higher-order harmonics) was used to collimate the polychromatic X-rays from a 2.9 T superbend magnet, which were subsequently monochromatized by a Si (111) channel-cut crystal. The monochromator was rocked at a frequency of 1 Hz, resulting in two spectra per second. A Rh-coated toroidal mirror focused on the beam. The Pd K-edge absorption spectra were collected in transmission mode using 15 cm long ionization chambers filled with 1 bar of Ar and 1 bar of N_2, and by measuring a Pd foil simultaneously for absolute energy calibration. Standard background subtraction, interpolation, and averaging were done with the Python-based software ProQEXAFS .[2] Normalization and analysis were performed with Larch.[3] The samples were measured in pellet form diluted by cellulose (1 : 0.5). The catalytic tests for Pd-PPCs are conducted using a batch and a flow reactor, and the products are analyzed with Gas Chromatography-Mass Spectrometry (GC-MS, PerkinElmer Clarus 560 gas choromatograph coupled to a Clarus 560 S mass spectrometer).

Synthetic Methods

Pd-pPPC 50 mg (0.281 mmol) of 1,2,4,5-tetracyanobenzene and 12.5 mg (0.07 mmol) of $PdCl_2$ and 625 mg (4.59 mmol) of $ZnCl_2$ are mixed and transferred into a Pyrex ampule (3 × 12 cm) under an inert atmosphere then we performed vacuum & Ar refill cycle three times at room temperature (the $ZnCl_2$ amount is back-calculated based on 2 wt % $PdCl_2$ in $ZnCl_2$). Then, the ampules were evacuated and sealed. The ampules were transferred into a box furnace and the temperature was raised to 300 °C (3 °C min^{-1} heating rate) and kept at this temperature for 3 hours, then further increased to 400 °C (3 °C min^{-1} heating rate) and kept under these conditions for 24 h in order to obtain Pd-pPPC. The ampule was cooled down to room temperature and opened carefully. The black crude of Pd-pPPC were ground into fine powders and washed thoroughly with water to remove $ZnCl_2$. Pd-pPPC was further stirred in 200 mL of DI water for 24 hours and then filtered. After this purification step, the resulting black powder was washed successively with 500 mL of DI water and then each 200 mL of acetone/EtOH. All powders were dried in a vacuum oven at 90 °C for 24 h.

Pd-pyPPC 50 mg (0.278 mmol) of 1,2,4,5-tetracyanopyrazine and 25 mg (0.14 mmol) of $PdCl_2$ and 1225 mg (8.98 mmol) of $ZnCl_2$ are mixed and transferred into a Pyrex ampule (3 × 12 cm) under an inert atmosphere then we performed vacuum & Ar refill cycle three times at room temperature. Then, the ampules were evacuated and sealed. The ampules were transferred into a box furnace and the temperature was raised to 300 °C (3 °C min^{-1} heating rate) and kept at this temperature for 3 hours, then further increased to 400 °C (3 °C min^{-1} heating rate) and kept it under these conditions for 24 h in order to obtain Pd-pyPPC. The ampule was cooled down to room temperature and opened carefully. The black crude of Pd-pyPPC was ground into fine powders and washed thoroughly with water to remove $ZnCl_2$. Pd-pyPPC was further stirred in 200 mL of DI water for 24 hours and then filtered. After this purification step, the resulting black powder was washed successively with 500 mL of DI water and then each 200 mL of acetone/EtOH. All powders were dried in a vacuum oven at 90 °C for 24 h.

Pd-pyPPC-NaCl 50 mg (0.278 mmol) of 1,2,4,5-tetracyanopyrazine and 25 mg (0.14 mmol) of $PdCl_2$, 1225 mg (8.99 mmol) of $ZnCl_2$ and 246 mg (4.21 mmol) of NaCl are mixed and transferred into a Pyrex ampule (3 × 12 cm) under an inert atmosphere then we performed vacuum & Ar refill cycle three times at room temperature. Then, the ampules were evacuated and sealed. The ampules were transferred into a box furnace and the temperature was raised to 300 °C (3 °C min^{-1} heating rate) and kept at this temperature for 3 hours, then further increased to 400 °C (3 °C min^{-1} heating rate) and kept it under these conditions for 24 h in order to obtain Pd-pyPPC-NaCl. The ampule was cooled down to room temperature and opened carefully. The black crude of Pd-pyPPC-NaCl was ground into fine powders and washed thoroughly with water to remove $ZnCl_2$. Pd-pyPPC-NaCl was further stirred in 200 mL of DI water for 24 hours and then filtered. After this purification step, the resulting black powder was washed successively with 500 mL of DI water and then each 200 mL of acetone/EtOH. All powders were dried in a vacuum oven at 90 °C for 24 h.

General catalyst test for Suzuki-Miyaura coupling reaction

A mixture of Pd-PPCs catalyst (10 mg), aryl bromide (1 mmol), R-phenyl boronic acid (1.2 mmol), base (2.4 mmol) solvent mixture (DMF : H_2O = 6 : 3 ml) was stirred at the required temperature for the required time to achieve the highest conversion under air. The reaction was monitored by GC-MS. After the

reaction was completed, the mixture was cooled down to room temperature extracted with dichloromethane (DCM) and water. The DCM phase was analyzed by using GC-MS to calculate the yields and selectivity.

General Set-up of Flow-Reactor

The continuous-flow Suzuki coupling reactions were conducted in a packed-bed reactor (Scheme S1). The designed packed-bed reactor (size 1.7 ml) was packed with 50 mg of catalyst. For the reaction solution, the aryl bromide (1 mmol), phenyl boronic acid (1.2 mmol) (or phenyl boronic acid ester), potassium carbonate (2.4 mmol) is dissolved in a mixture of organic solvent (DMF 6 ml) and water (3 ml), and the mixture was pumped into packed-bed reactor at a flow rate of 0.1 ml min^{-1} and 120 ^{o}C. (These flow rate and reaction temperature are determined through optimization experiments (Figure S8 – 10). In each reaction condition, the sample solution was collected and analyzed with GC-MS.

ICP-OES sample preparation and metal content determination

For ICP-OES analysis, samples (pyPPC–NaCl, pyPPC, and pPPC) were thermally treated by TGA at 1000 °C under air to remove the organic framework. The resulting residues were digested in 2.5 mL of freshly prepared aqua regia (HNO_3:HCl = 1:3, v/v) and left to stand for 10 days to ensure complete dissolution. Sample solutions were prepared by diluting 0.3 mL of the digested mixture with 9.7 mL of double deionized (DDI) water. A blank solution was prepared by diluting 0.3 mL of aqua regia with 9.7 mL of DDI water. Calibration standards were prepared by serially diluting a 1000 ppm Pd or Zn stock solution to concentrations of 200, 120, 50, 20, 5, and 0.5 ppm. The measurements were performed using an ICP-OES instrument. The resulting elemental concentrations were used to calculate the Pd and Zn weight percentages in the original polymer samples. All calculations were performed using Microsoft Excel.

Structural analysis of Pd-pPPC and Pd-PyPPC-NaCl

Crystal models were simulated with the use of Biovia Materials Studio Software. Structures composed of square layers (**sql**), formed by connecting Pd-phthalocyanine units were modeled, and geometrically optimized via energy minimization procedures employing the universal force field implemented in the Forcite module. A structure with layers stacked in a nearly eclipsed configuration exhibited the best agreement between the calculated and experimental PXRD patterns. In this model, belonging to the orthorhombic *Cmcm* space group there are two layers per unit cell, with interlayer distance of 3.4 Å. Atomic coordinates for both structures are provided (Table S5 and 6).

Computational methods

Density Functional Theory (DFT) calculations were performed using the Vienna *Ab initio* Simulation Package (VASP).[4-5] The exchange-correlation energies were described within the generalized gradient approximation (GGA) using the Perdew-Burke-Ernzerhof (PBE) functional, with dispersion interactions incorporated via Grimme's DFT-D3 correction.[6-7] The projected-augmented wave (PAW) method was employed to describe core electrons, while valence electrons were expanded in a plane-wave basis set with a cutoff energy of 450 eV. Spin polarization was included when necessary.[8-9] Structural relaxations were performed until the total energy converged to within 10^{-4} eV for ionic relaxation and 10^{-5} eV for

electronic self-consistency. For slab models, a dipole correction was applied along the *z*-axis to mitigate asymmetry effects.[10] The Covalent Organic Framework (COF) was modelled with a single unit cell containing a C-N backbone and one Pd atom per unit cell. To explore the relaxation pathway a 2x2 supercell was employed. No additional layer was considered for the reaction mechanism. Two coordination sites were studied: i) the cavity, defined as a site surrounded by four nitrogen terminations, and ii) the pore, which arises from the linkage of four COF units. Transition states were located by using Climbing Image Nudge Elastic Band (CI-NEB). They were verified through a frequency numerical analysis with a step size of ± 0.015 Å and confirmed by the presence of only one imaginary frequency.[11] A data set collection of computational results is available in the ioChem-BD repository[12] and can be accessed via https://doi.org/10.19061/iochem-bd-1-377.

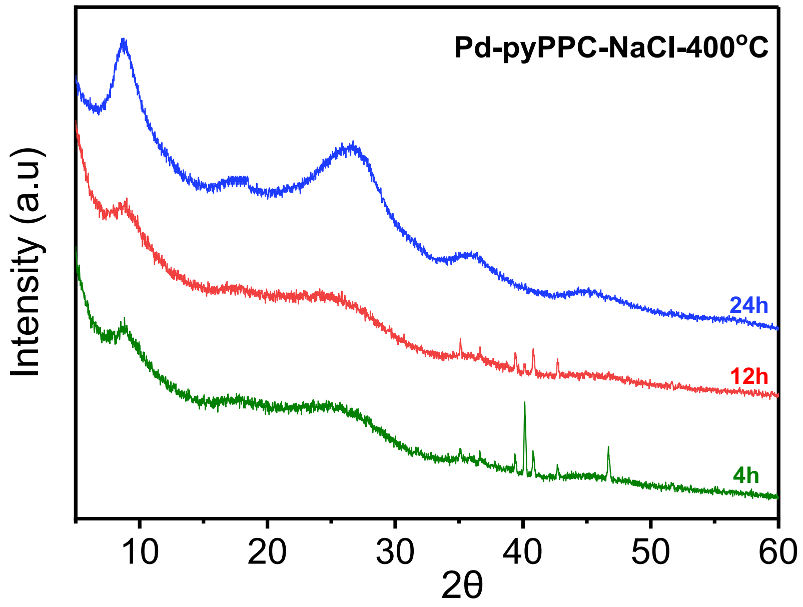

Figure S1. PXRD patterns of Pd-pyPPC-NaCl prepared at 400 °C for 4 h, 12 h, and 24 h.

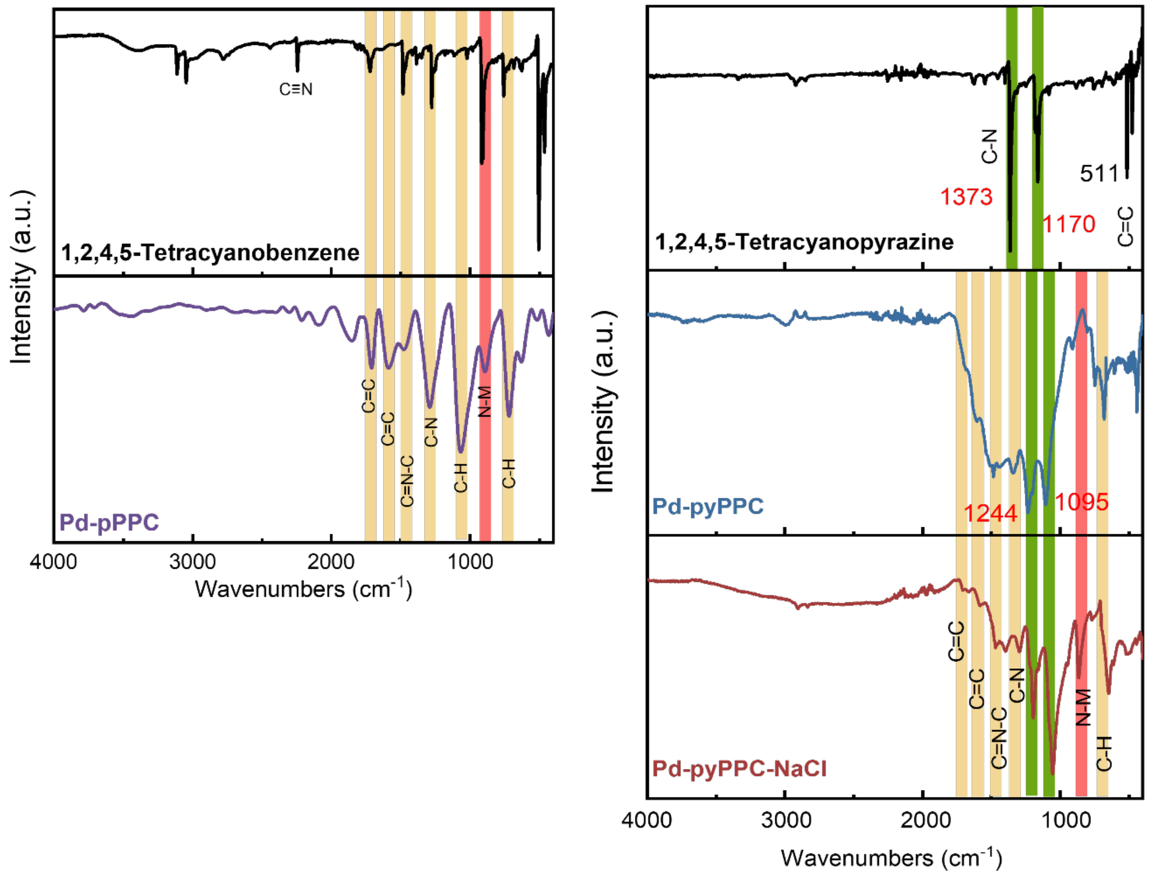

Figure S2. FT-IR analysis of precursors and Pd-PPCs.

Table S1. Metal interchange from Zn to Pd. Reference is Zn-pyPPC on different positions + $PdCl_2$.

METAL INTERCHANGE			
ΔE (Zn-pyPPC + $PdCl_2$ → Pd-pyPPC + $ZnCl_2$)			
**Ads. site**	**M**	**MCl_2***	**$2H^*_{cav}$ + MCl_2**
M_{Cav}	-3.54	-2.67	-
M_{Pore}	-2.30	-1.40	-1.02

Table S2. Elemental analysis (EA) and Inductively Coupled Plasma Optical Emission spectroscopy (ICP-OES) results of Pd-PPCs.

Computed value[a]			
	**Pd-pPPC**	**Pd-pyPPC**	**Pd-pyPPC-NaCl**
**C**	51.91	41.18	41.18
**N**	24.22	36.02	36.02
**H**	0.87	-	-
**Pd**	23	22.8	22.8
**Total**	100	100	100
**Experimental value**			
	**Pd-pPPC**	**Pd-pyPPC**	**Pd-pyPPC-NaCl**
**C[b]**	47.3	36.6	36.3
**N[b]**	18.3	27.7	28.6
**H[b]**	1.5	2.1	1.3
**Pd[c]**	19.2	22.2	18.3
**Total**	86.3	88.6	84.5

[a] Computed values based on the pristine pure unit cell of each catalyst and an average of 1 Pd atom per unit: Pd-pPPC ($C_{20}H_4N_8Pd$), Pd-pyPPC ($C_{16}H_4N_{12}Pd$) and Pd-pyPPC-NaCl ($C_{16}H_4N_{12}Pd$), [b] Measured by EA, [c] Measured by ICP-OES

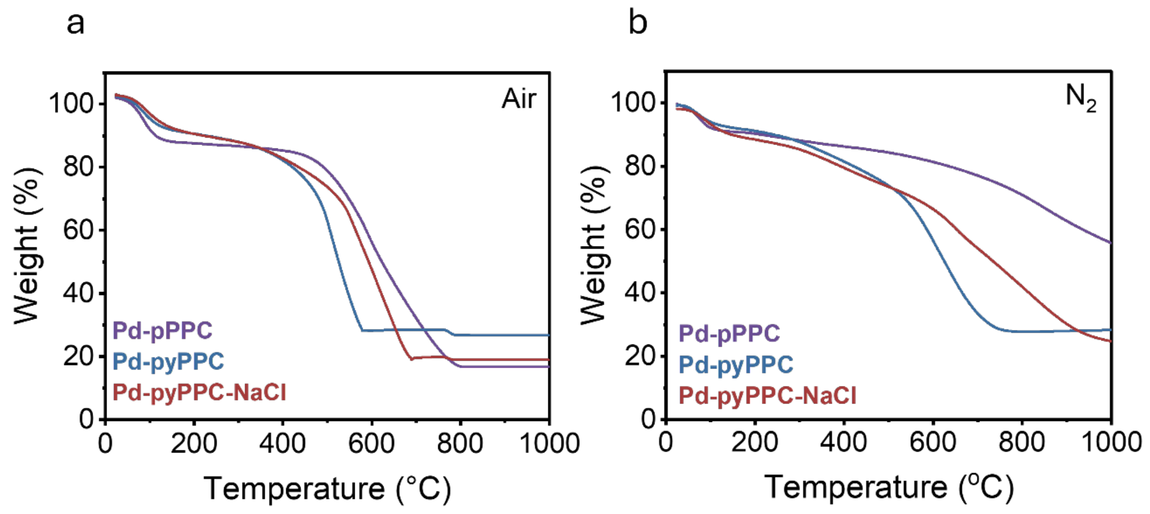

Figure S3. TGA analysis of Pd-PPCs in (a) air and (b) nitrogen.

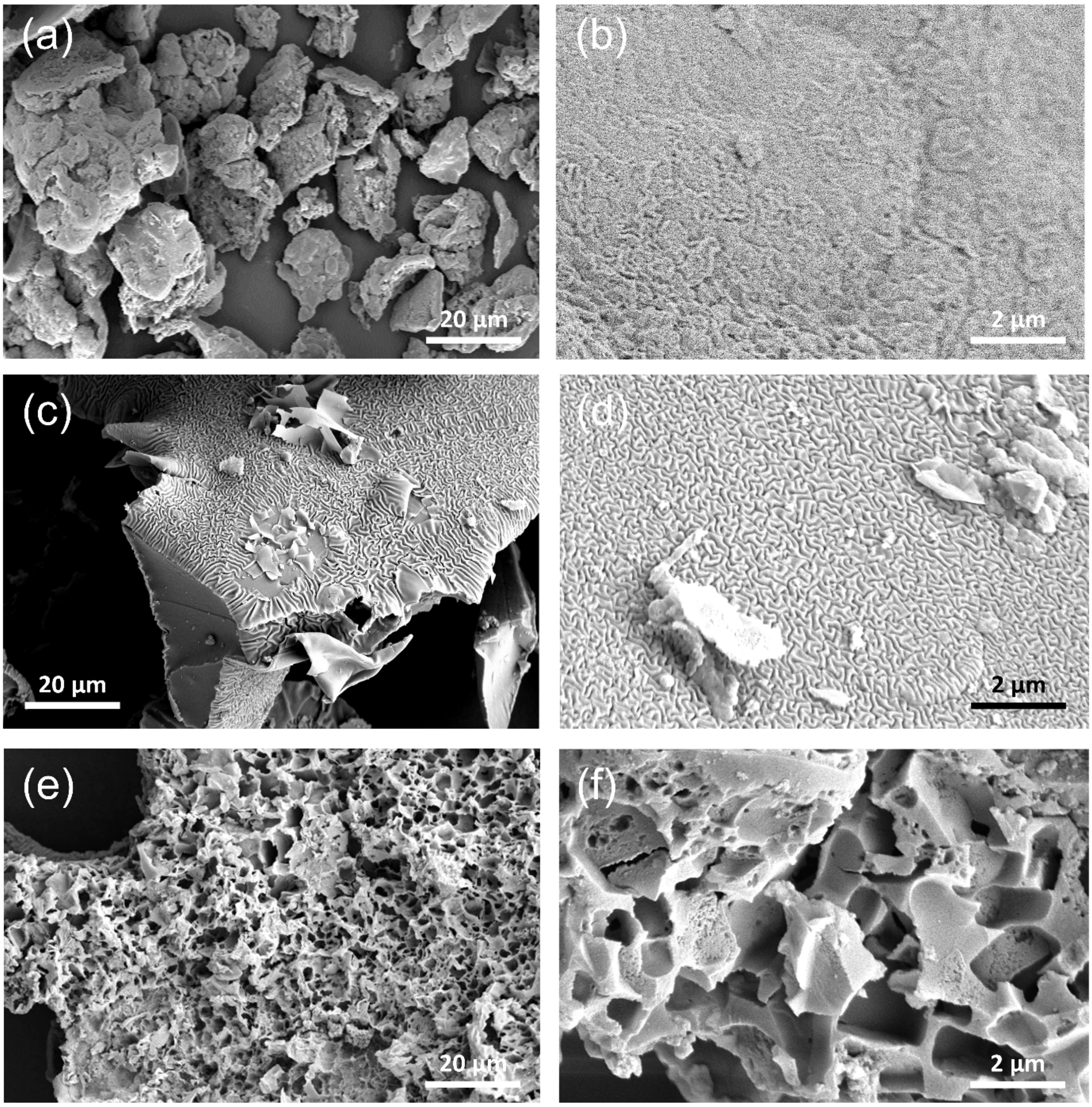

Figure S4. SEM analysis of (a), (b) Pd-pPPC, (c), (d) Pd-pyPPC and (e), (f) Pd-pyPPC-NaCl

Table S3. Atomic coordinates of Pd-PPC optimized crystal model
Compound: Pd-pPPC
Latice parameters: Orthorhombic
Space group (H–M): Cmcm
Space group number: 63
a = 14.8987 Å, b = 14.8987 Å, c = 6.8155 Å
α = 90.0000°, β = 90.0000°, γ = 90.0000°

Atom name	Fractional coordinates		
	x	y	z
Pd1	0.00000	0.04277	0.25000
N2	0.00000	0.81910	0.25000
N3	0.00000	0.26641	0.25000
N4	0.27633	0.54275	0.25000
N5	-0.09331	0.13605	0.25000
N6	0.40672	0.44947	0.25000
C7	-0.07949	0.22506	0.25000
C8	-0.16259	0.27080	0.25000
C9	0.31770	0.46328	0.25000
C10	0.27197	0.38019	0.25000
C11	0.31767	0.62223	0.25000
C12	0.27192	0.70531	0.25000
C13	-0.18136	0.36144	0.25000
C14	0.07946	0.86046	0.25000
C15	0.16254	0.81470	0.25000
C16	0.18129	0.72407	0.25000
H17	0.37000	0.08718	0.75000
H18	0.12992	0.32730	0.75000

Table S4. Atomic coordinates of Pd-PPC-NaCl optimized crystal model

Compound: Pd-PyPPC-NaCl
Lattice parameters: orthorhombic
Space group (H-M): Cmcm
Space group number: 63
a = 14.8987 Å b = 14.8987 Å c = 6.8156 Å
α = 90.0000°, β = 90.0000°, γ = 90.0000°

Atom name	Fractional coordinates		
	x	y	z
Pd1	-0.00000	0.03855	0.25000
N2	0.00000	0.81383	0.25000
N3	0.00000	0.26325	0.25000
N4	0.27526	0.53853	0.25000
N5	-0.09392	0.13244	0.25000
N6	0.40612	0.44466	0.25000
C7	-0.07984	0.22192	0.25000
C8	-0.16368	0.26748	0.25000
C9	0.31663	0.45871	0.25000
C10	0.27108	0.37489	0.25000
C11	0.31659	0.61836	0.25000
C12	0.27101	0.70217	0.25000
N13	-0.18226	0.35630	0.25000
C14	0.07981	0.85517	0.25000
C15	0.16361	0.80958	0.25000
N16	0.18219	0.72076	0.25000

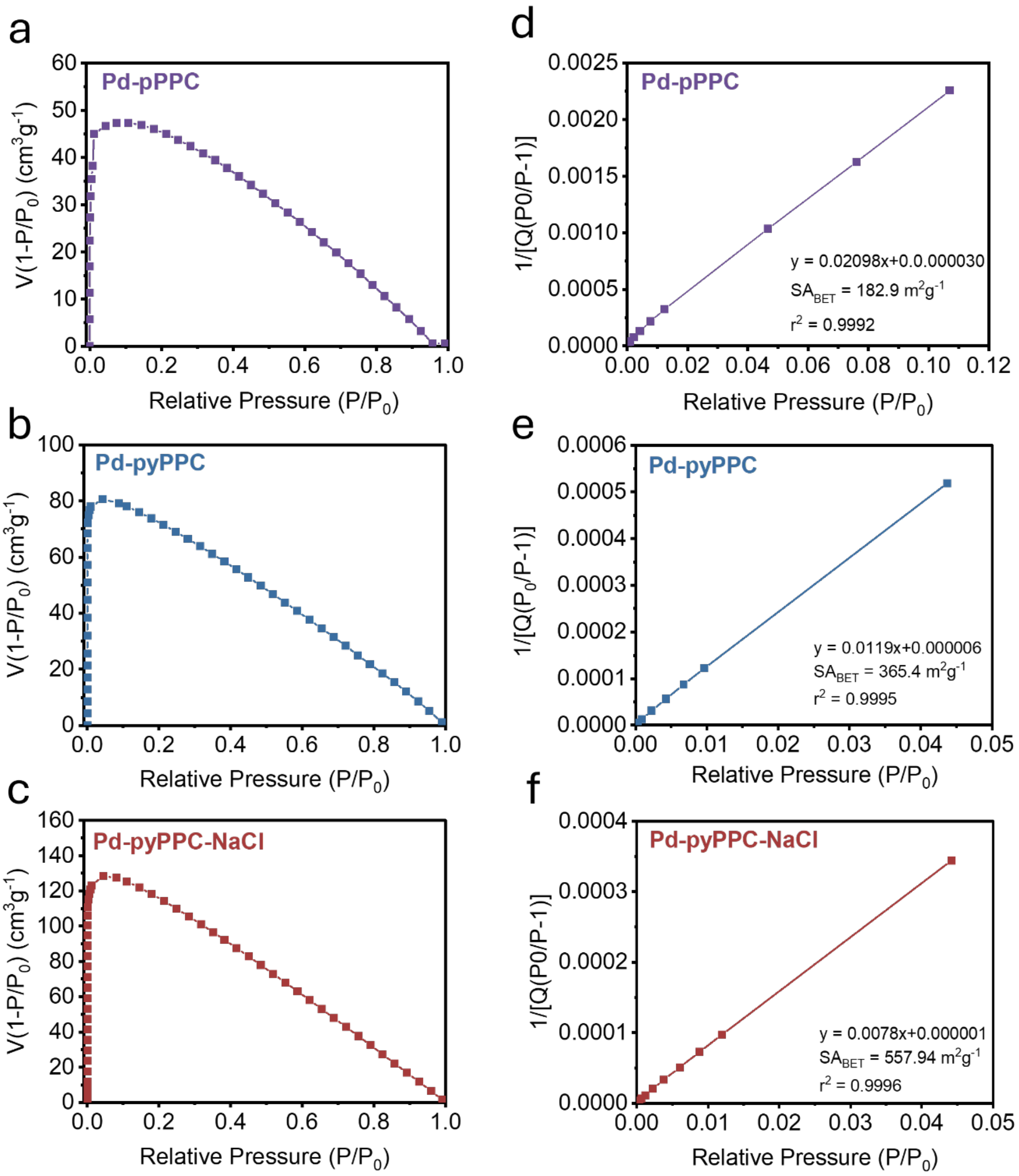

Figure S5. Rouquerol plots for (a) Pd-pPPC, (b) Pd-pyPPC, and (c) Pd-pyPPC–NaCl, and BET linear plots for (d) Pd-pPPC, (e) Pd-pyPPC, and (f) Pd-pyPPC–NaCl.

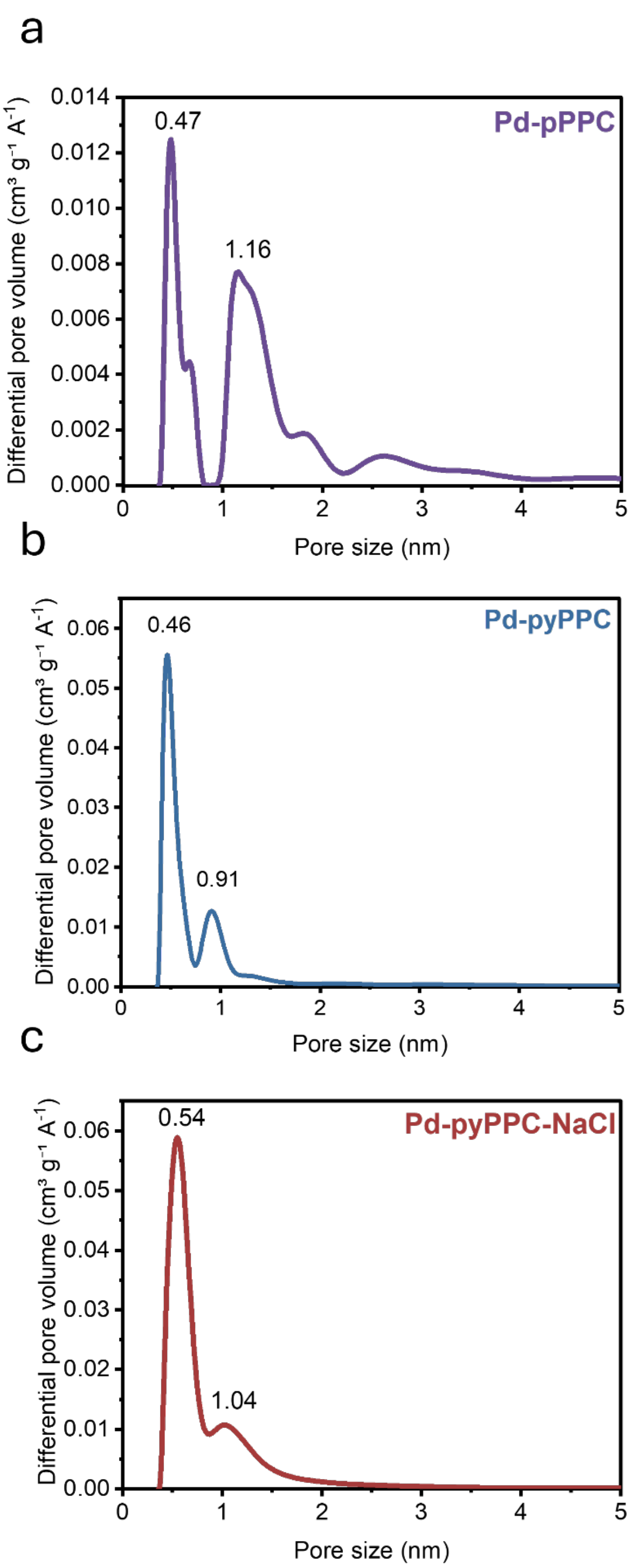

Figure S6. NLDFT pore size distribution (PSD) analysis of a) Pd-pPPC, b) Pd-pyPPC, c) Pd-pyPPC-NaCl

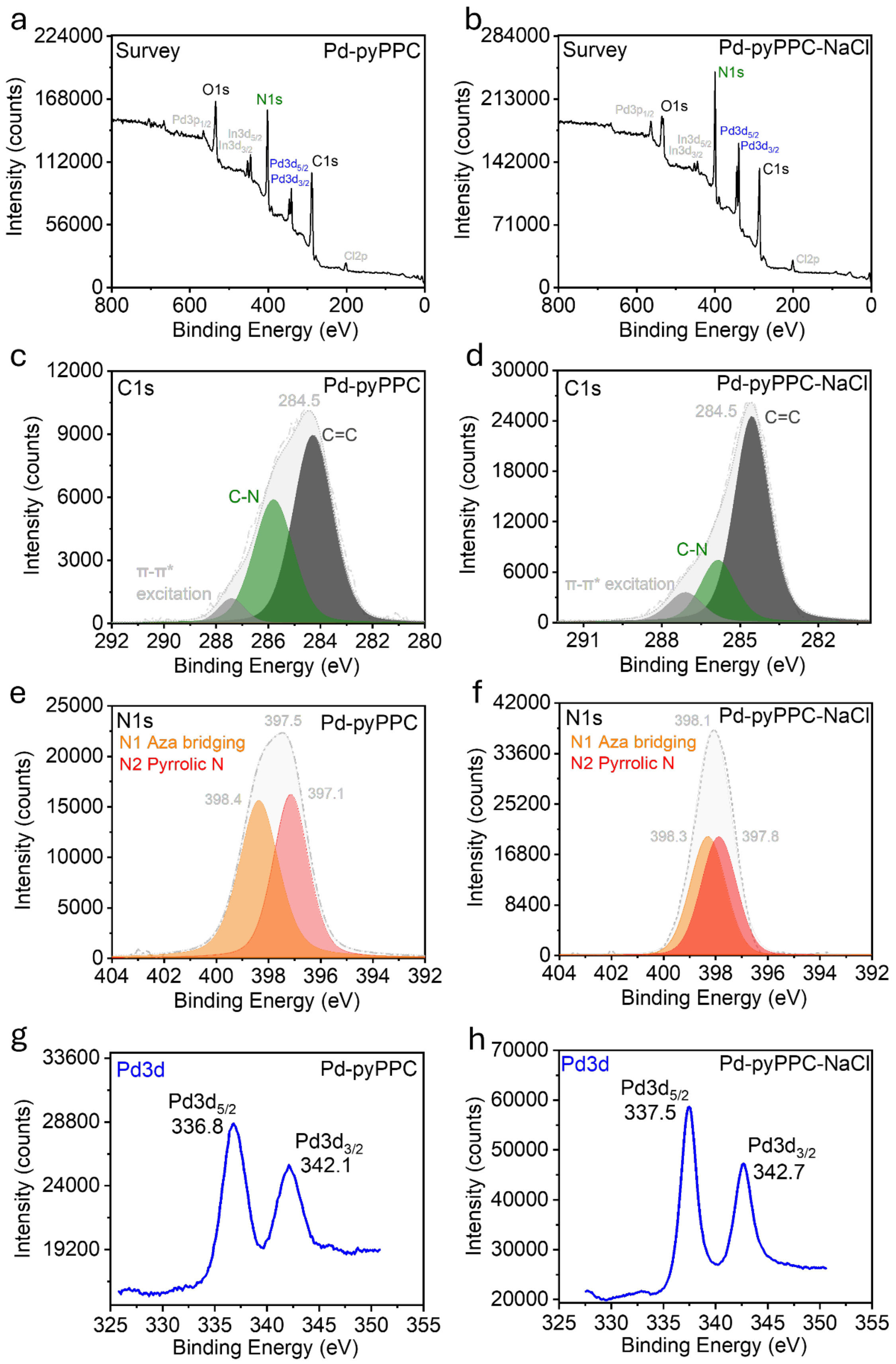

Figure S7. XPS analysis of Pd-pyPPC ((a), (c), (e), (g)) and Pd-pyPPC-NaCl ((b), (d), (f), (h))

Table S5. Dechlorination pathway for the two different adsorption sites of Pd on the pyPPC COF. The reference state is the COF with two H atoms adsorbed and $PdCl_2$(g).

DECHLORINATION PATHWAY				
	ΔG of each intermediate / eV			
**Ads. site**	**2H* + $PdCl_2$**	**$PdCl_2$* + 2H***	**PdCl +HCl(g)**	**Pd + 2HCl(g)**
Pd_{cav}	0.00	1.13[1]	-1.79	-3.06
Pd_{pore}	0.00	-1.99	-0.23	2.71

[1]When Pd is adsorbed at Pd_{cav}, both H* are expelled as H_2(g).

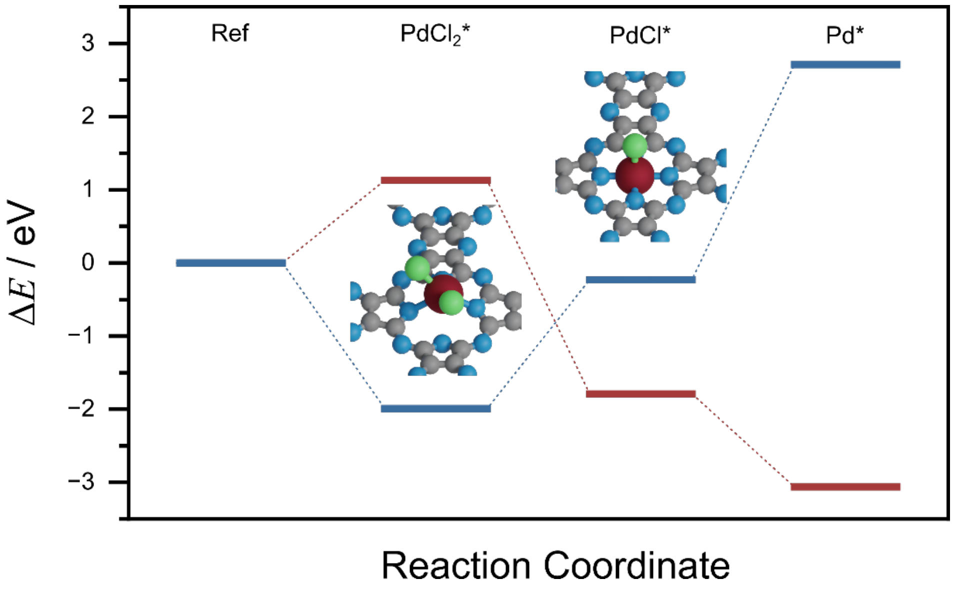

Figure S8. Gibbs free energies of the dechlorination pathway for the formation of the single atom catalysts on the two different adsorption sites. There are two images of the two intermediates on Pd_{cav}. All structures can be found at the ioChem-BD repository (https://doi.org/10.19061/iochem-bd-1-377). Color code: C: gray, N: light blue, Pd:red, Cl: green.

Table S6. Interlayer distances (Å) from L1(bottom layer) to L3(top layer) for Pd_X-pyPPC COF. Inter refers to interlayer of L2-L3.

NUMBER OF PD	POSITIONS	L1-L2	L2-L3
**0Pd**		3.63	3.60
**1Pd$_{cav}$**	L1	3.58	3.60
	L2	3.60	3.63
	L3	3.67	3.59
	Inter[1]	3.64	3.26
**1 Pd$_{pore}$**	L1	3.51	3.58
	L2	3.49	3.49
	L3	3.59	3.50
	inter	3.56	3.60
**2Pd$_{cav}$**	L1 L2	3.65	3.60
	L1 L3	3.60	3.60
	L1 inter[1]	3.59	3.25
**2Pd$_{pore}$**	L1 L2	3.48	3.48
	L1 L3	3.47	3.48
	L1 inter	3.53	3.62
**Pd$_{cav}$ Pd$_{pore}$**	L1 L1	3.57	3.60
	L1 L2	3.57	3.59
	L1 L3	3.64	3.62
	inter L2	3.56	3.58
	L2 L3	3.48	3.48
	L2 L2	3.50	3.48
**3 Pd$_{cav}$**	L1 L2 L3	3.65	3.66

**$2Pd_{cav}$ $1Pd_{pore}$**	L1 L3 inter	3.55	3.48
**$3Pd_{cav}$ $1Pd_{pore}$**	L2	3.57	3.56
	L3	3.63	3.52
	inter (2bonds)[2]	3.64	3.62
**$1PdCl_{2pore}$**	L3	3.61	3.59
	inter (2bonds)[2]	3.56	3.49
**$3Pd_{cav}$ $PdCl_{2pore}$**	inter (2bonds)[2]	3.68	3.53

[1]inter cavity is 3 eV above their cavity analogous; [2] Pd bonded in the interlayer only to two nitrogen atoms.

Table S7. Base screening test of Pd-pyPPC.

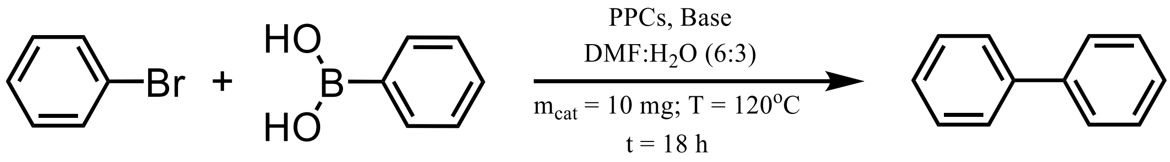

Entry	Base	Yield (%)
**1**	Na_2CO_3	21
**2**	K_2CO_3	90
**3**	Cs_2CO_3	60
**4**	NaOH	57
**5**	KOH	25
**6**	TEA	16

Table S8. Catalytic performance of Pd-PPCs for Suzuki-Miyaura cross-coupling in batch reaction.

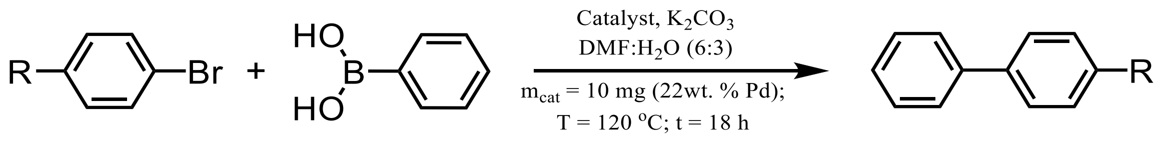

Entry	Catalyst	R	Yield (%)
**1**	Pd-pPPC	CH_3	32
**2**	Pd-pPPC	OCH_3	39
**3**	Pd-pPPC	CN	50
**4**	Pd-PyPPC	CH_3	51
**5**	Pd-PyPPC	OCH_3	74
**6**	Pd-PyPPC	CN	89
**7**	Pd-PyPPC-NaCl	CH_3	35
**8**	Pd-PyPPC-NaCl	OCH_3	62
**9**	Pd-PyPPC-NaCl	CN	83
**10**	$Pd(PPh_3)_4$	CH_3	70
**11**	$Pd(PPh_3)_4$	OCH_3	86
**12**	$Pd(PPh_3)_4$	CN	87
**13**	Pd/C	CH_3	76
**14**	Pd/C	OCH_3	74
**15**	Pd/C	CN	85

Table S9. Catalytic performance of Pd-PC model compound for Suzuki-Miyaura cross-coupling under batch and flow reaction.

Sample	Yield (%)
Fresh Pd-Pc (batch)	24.4
Reused Pd-Pc (2nd cycle batch)	5.9
Pd-Pc in flow	9.4

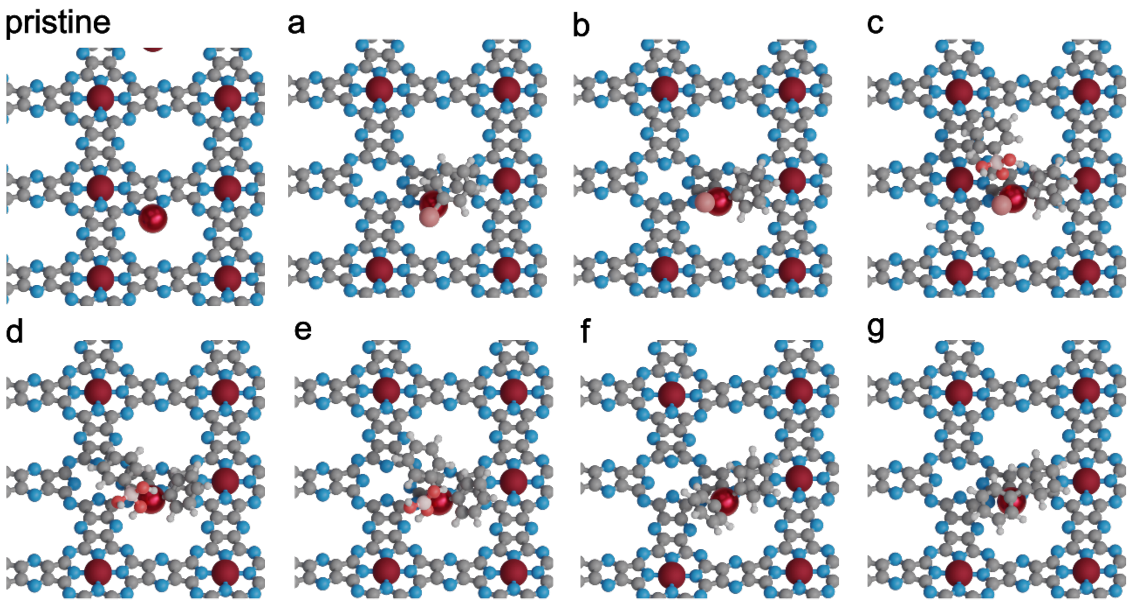

Figure S9. Illustration of the intermediates of the reaction pathway on Pd-pyPPCs, a-g corresponding to the intermediates collected in Supporting Table 9. Color code: O: red, B: light brown, C: gray, N: light blue, H: light gray, Pd_{Cav}: dark red, Pd_{Pore}: metallic red.

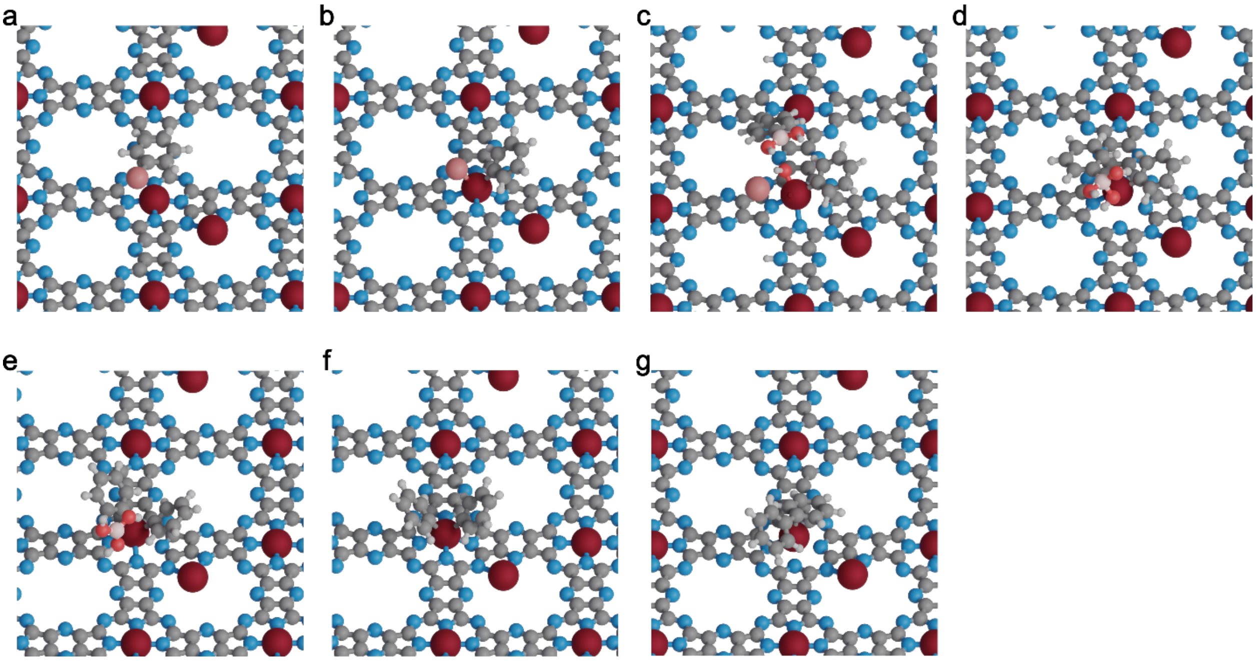

Figure S10. Illustration of the intermediates of the reaction pathway on Pd_{cav}-pyPPCs, a-g corresponding to the list of intermediates collected in Supporting Table 9. Color code: O: red, B: light brown, C: gray, N: light blue, H: light gray, PdCav: dark red, PdPore: metallic red.

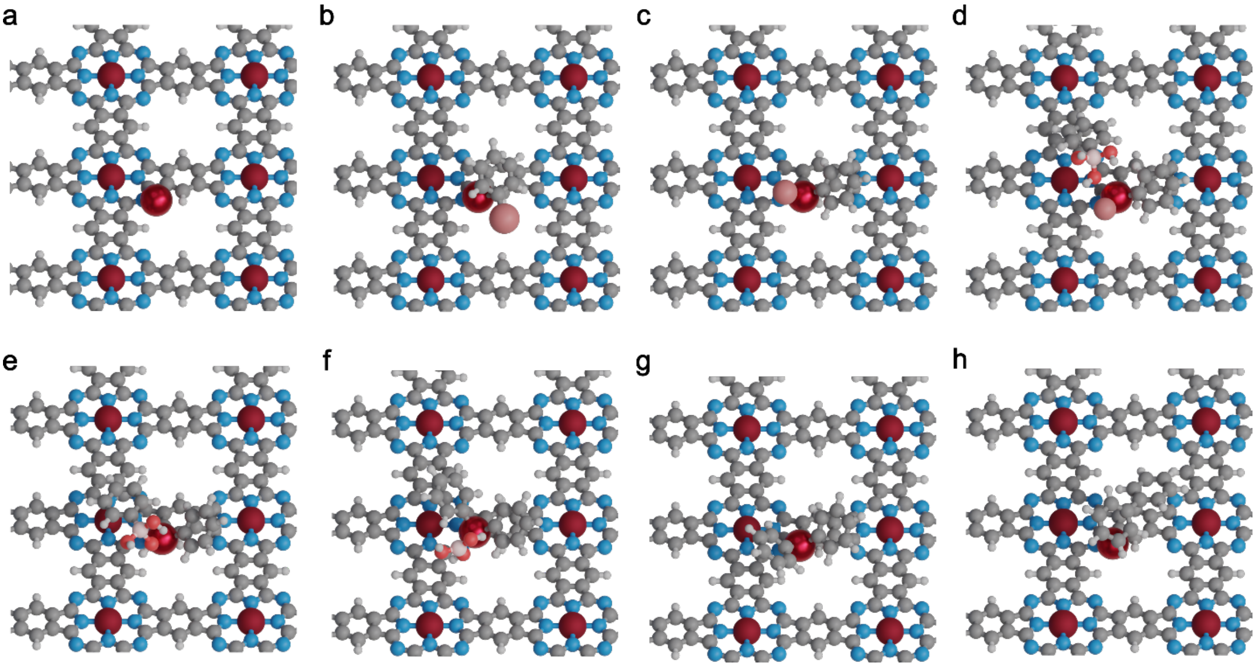

Figure S11. Illustration of the intermediates of the reaction pathway on Pd-pPPCs, a being the pristine one, and b-h corresponding to the intermediates collected in Supporting Table 9. Color code: O: red, B: light brown, C: gray, N: light blue, H: light gray, Pd_{Cav}: dark red, Pd_{Pore}: metallic red.

Table S10. Relative energies (ΔE, in eV) of the key intermediates and transition states involved in the reaction pathway. Energies are referenced to the pristine surface with the isolated reactants (ΔE = 0.00 eV). The last column is the core level electron shift energies with the pristine Pd^{2+}* as reference.

Intermediates	Gas Phase	ΔE / eV	ΔBE / eV
Pristine + PhBr + $PhB(OH)_2$ + H_2O	0.00	0.00	0.00
PhBr* + $PhB(OH)_2$ + H_2O (a)	-1.24	-1.09	0.5
TS1	0.04	-0.92	-
Ph* + Br* + $PhB(OH)_2$ + H_2O (b)	-0.49	-1.75	2.4
Ph* + Br* + $PhB(OH)_3^*$ + H^* (c)	-	-1.25	2.3
Ph* + $PhB(OH)_3$* + HBr(g) (d)	0.50	-1.45	2.6
TS2	0.80	-0.73	-
Ph* + $B(OH)_3$* Ph* (e)	-1.90	-2.03	2.4
Ph* Ph* + $B(OH)_3$ (g) (f)	-1.03	-1.82	2.6
TS3	-0.48	-1.50	-
Ph-Ph* (g)	-2.90	-3.03	0.5
Pristine + Products	-1.40	-1.40	

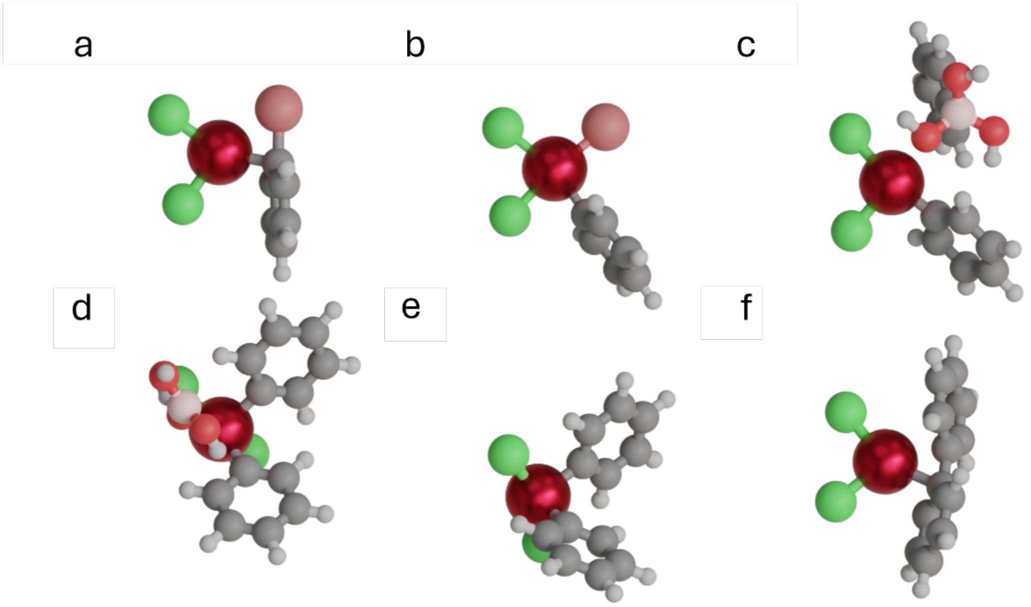

Figure S12. Illustration of the intermediates of the reaction pathway on a released $PdCl_2$, a-g corresponding to the intermediates collected in Supporting Table 9. C is missing in order to match the species involved in the COF catalysts. Color code: Cl: green, O: red, B: light brown, C: gray, N: light blue, H: light gray, Pd: metallic red.

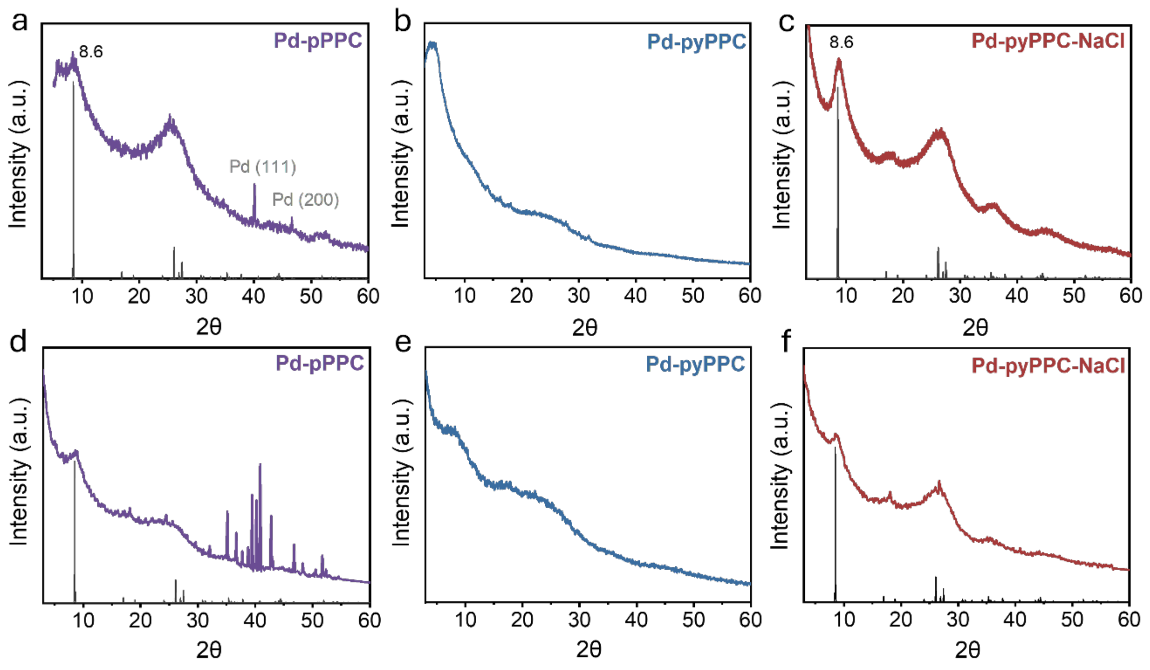

Figure S13. PXRD comparison (a), (b), (c) before and (d), (e), (f) after Suzuki-Miyaura cross-coupling reaction.

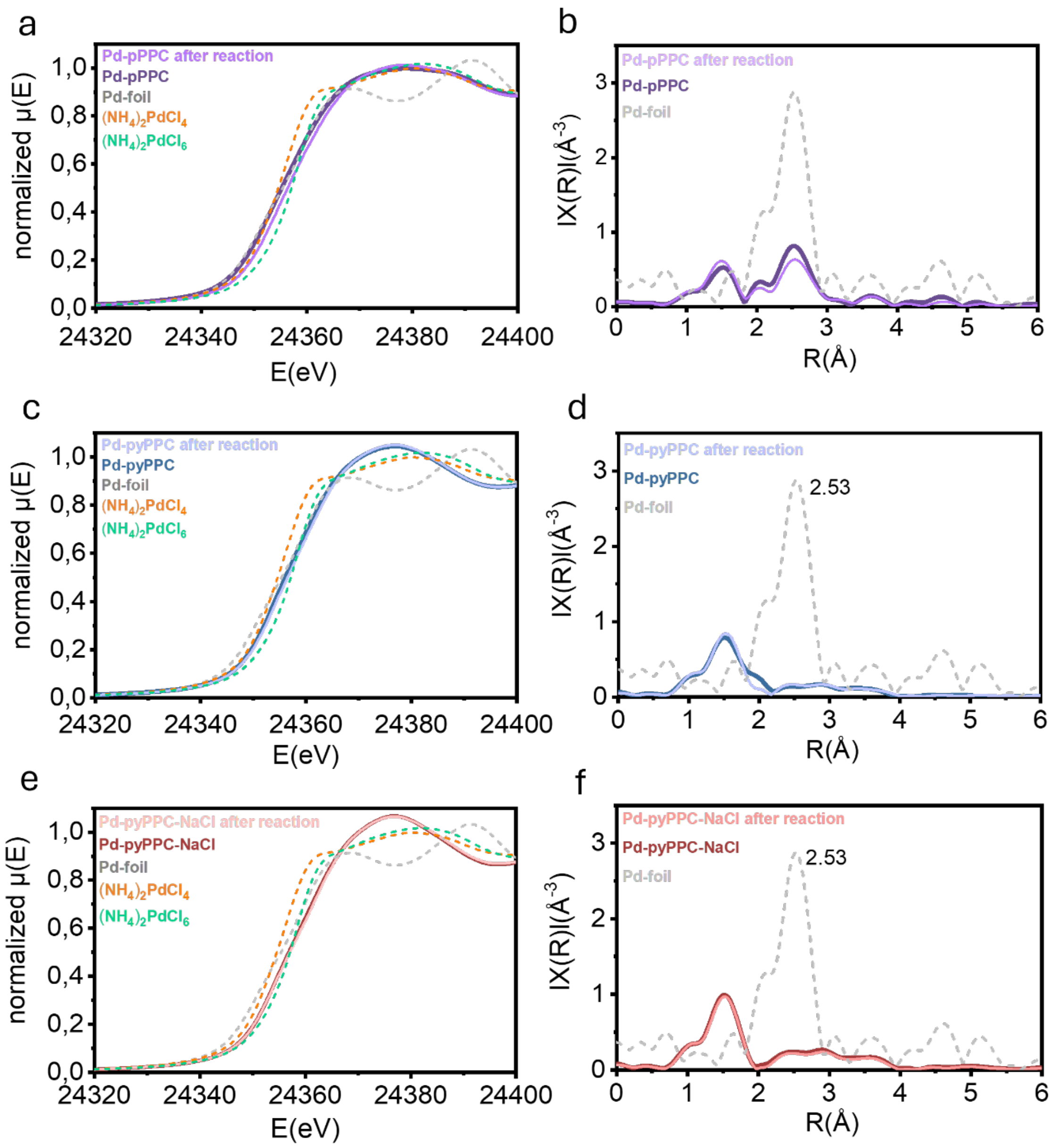

Figure S14. Comparison of X-ray absorption near edge structure (XANES) of Pd-PPCs and R-space analysis before and after cross-coupling reaction.

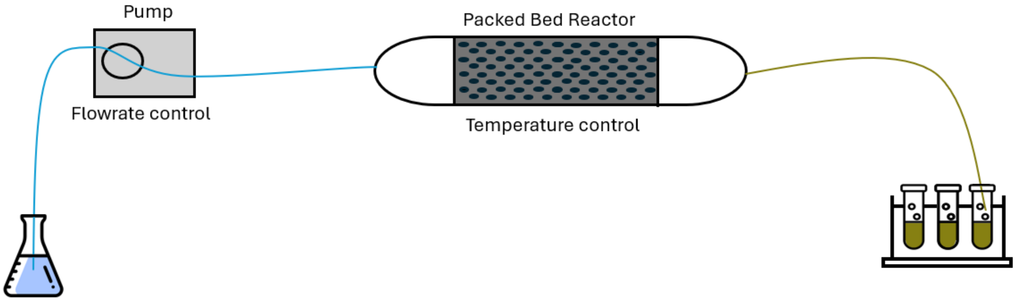

Supplementary Scheme 1 | Schematic illustration of the flow reactor.

Table S11. Investigation of flow rate and temperature effect on the continuous Suzuki-Miyaura cross-coupling reaction of Pd-pyPPC.

Temperature (°C)	Flow rate (ml min^{-1})	Yield (%)
**100**	0.05	27
	0.1	77
	0.2	46
	0.4	31
	0.6	9
**120**	0.05	39
	0.1	90
	0.2	69
	0.4	48
	0.6	28
**140**	0.05	46
	0.1	73
	0.2	75
	0.4	52
	0.6	45
**160**	0.05	47
	0.1	63
	0.2	62
	0.4	56
	0.6	62

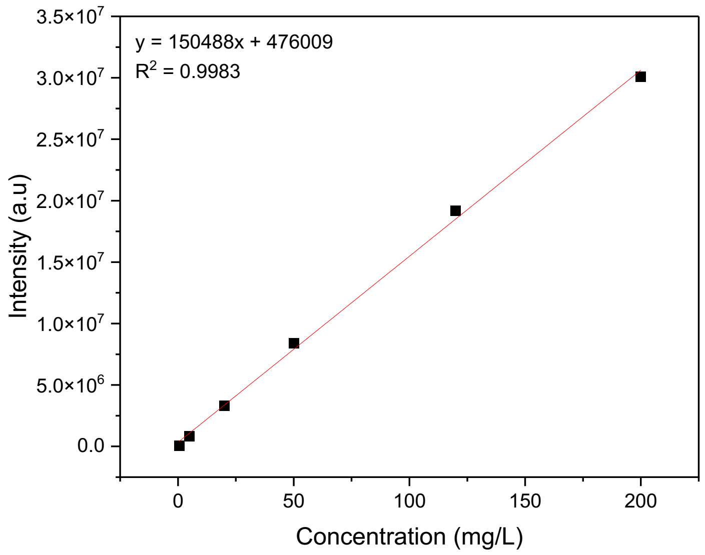

Figure S15. ICP-OES calibration curve used for Pd quantification after 24-hour time-on-stream Suzuki-Miyaura cross-coupling flow reactions.

Table S12. Pd quantification results for Pd–pyPPC–NaCl and Pd/C catalysts after 24-hour time-on-stream Suzuki-Miyaura cross-coupling reactions.

Catalyst	Intensity	Concentration (mg/L)	Digested volume (L)	Volume used (L)	Diluted volume (L)	Dilution factor	Sample mass (mg)	Pd wt %
**Pd-pyPPC-NaCl**	4794161.9	28.7	0.0025	0.0003	0.01	33.33	13.2165	18.2
**Pd/C**	329665.2	-0.97	0.0025	0.0003	0.01	33.33	12.4352	n.d.

References

[1] T. G. Witkowski, E. Sebastiao, B. Gabidullin, A. Hu, F. Zhang, M. Murugesu, *ACS Appl. Energy Mater.* **2018**, *1*, 589-593.

[2] A. H. Clark, J. Imbao, R. Frahm, M. Nachtegaal, *J. Synchrotron Radiat.* **2020**, *27*, 551-557.

[3] M. Newville, *J. Phys.: Conf. Ser.* **2013**, *430*, 012007.

[4] G. Kresse, J. Furthmüller, *Comput. Mater. Sci.* **1996**, *6*, 15-50.

[5] G. Kresse, J. Furthmüller, *Phys. Rev. B* **1996**, *54*, 11169-11186.

[6] J. P. Perdew, K. Burke, M. Ernzerhof, *Phys. Rev. Lett.* **1996**, *77*, 3865-3868.

[7] S. Grimme, J. Antony, S. Ehrlich, H. Krieg, *J. Chem. Phys.* **2010**, *132*, 154104.

[8] G. Kresse, D. Joubert, *Phys. Rev. B* **1999**, *59*, 1758-1775.

[9] P. E. Blochl, *Phys. Rev. B Condens. Matter.* **1994**, *50*, 17953-17979.

[10] G. Makov, M. C. Payne, *Phys. Rev. B Condens. Matter.* **1995**, *51*, 4014-4022.

[11] G. Henkelman, B. P. Uberuaga, H. Jónsson, *J. Chem. Phys.* **2000**, *113*, 9901-9904.

[12] M. Alvarez-Moreno, C. de Graaf, N. Lopez, F. Maseras, J. M. Poblet, C. Bo, *J. Chem. Inf. Model.* **2015**, *55*, 95-103.
